# Supplementary material for: UniCAS: A foundation model for cervical cytology screening
Source: Cell Rep Med. 2026 Jan 20;7(1):102570. doi: 10.1016/j.xcrm.2025.102570 (PMC12866161; doi:10.1016/j.xcrm.2025.102570)
Supplement: Document S1. Figures S1 and S2 and Tables S1–S20 [file mmc1.pdf]

Cell Reports Medicine, Volume *7*

Supplemental information

UniCAS: A foundation model for cervical cytology screening

Haotian Jiang, Jiangdong Cai, Zhenrong Shen, Mengjie Xu, Manman Fei, Haolin Huang, Xinyu Wang, Rui Bi, Dinggang Shen, Lichi Zhang, and Qian Wang

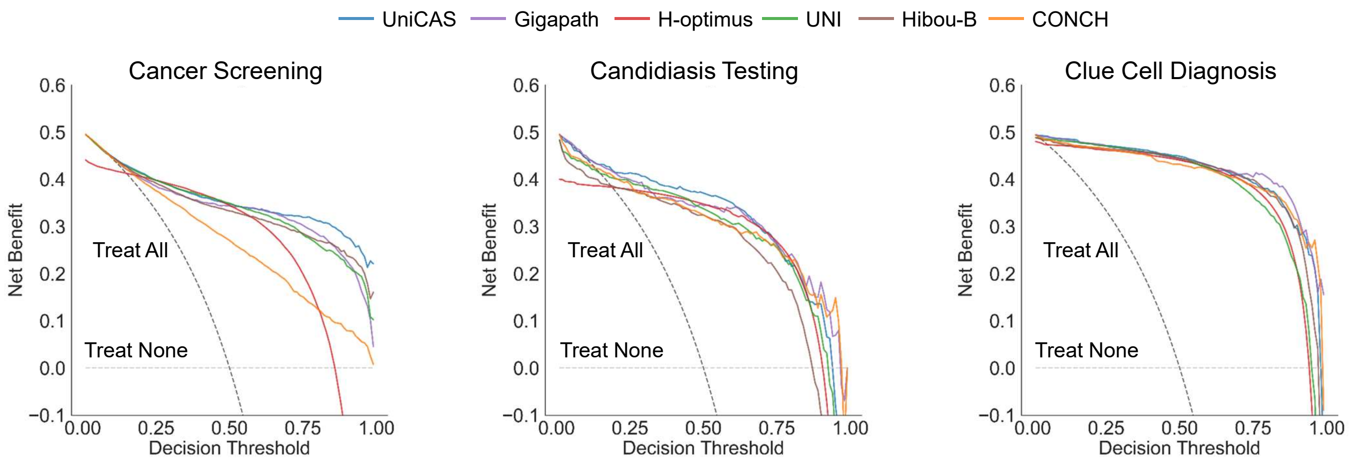

Figure S1. Decision curve for slide-level diagnosis. A model is considered clinically useful if its curve lies above both the "Treat All" and "Treat None" strategies. The curve that lies above others indicates the greatest net benefit. Related to Figure 2.

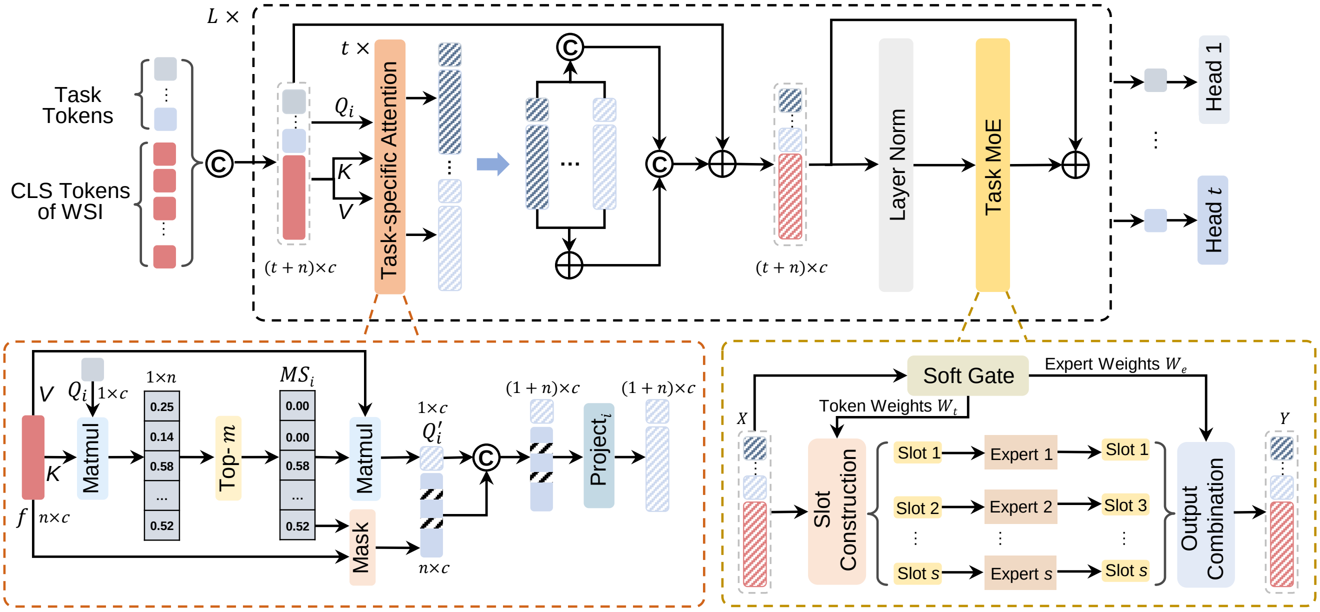

Figure S2. Overview of the multi-task aggregator (top), with Task-specific Attention and Task Mixture of Experts (MoE) zoomed in (bottom). Related to STAR Methods.

Table S1. Age distribution of pre-training data. Related to Figure 1.

**Ages**	**Number of Slides**
15–20	296
21–25	1,635
26–30	3,758
31–35	7,729
36–40	8,024
41–45	6,936
46–50	6,112
51–55	5,827
56–60	4,217
61–65	2,022
66–70	1,174
71–75	545
76–80	193
81–85	50
86–90	14
Total	48,532

Table S2. Comparison of slide-level diagnosis results using TransMIL (%, 95% CI). Related to Figure 2.

Task	Encoder	AUC	F1-score	Sensitivity	Specificity
Cancer Screening	Gigapath	90.32 (89.53–91.11)	82.96 (81.95–83.98)	81.58 (80.15–83.01)	84.68 (83.41–85.95)
	H-optimus	89.86 (89.06–90.66)	82.56 (81.56–83.57)	82.93 (81.51–84.35)	82.14 (80.78–83.49)
	UNI	89.96 (89.18–90.75)	82.50 (81.38–83.62)	80.41 (78.86–81.96)	**85.45 (84.19–86.71)**
	Hibou-B	88.40 (87.55–89.24)	80.81 (79.69–81.92)	80.44 (79.09–81.79)	81.40 (80.10–82.71)
	CONCH	82.35 (81.31–83.39)	75.12 (73.96–76.28)	75.03 (73.44–76.61)	75.25 (73.61–76.88)
	**UniCAS**	**90.90 (90.12–91.68)**	**83.70 (82.65–84.75)**	**83.37 (82.01–84.73)**	84.10 (82.79–85.40)
Candidiasis Testing	Gigapath	90.77 (87.40–94.14)	83.12 (78.69–87.54)	82.48 (76.58–88.39)	83.88 (78.00–89.76)
	H-optimus	90.26 (86.84–93.68)	83.29 (78.85–87.73)	**83.16 (76.92–89.40)**	83.54 (77.78–89.31)
	UNI	90.44 (86.99–93.88)	82.34 (77.73–86.96)	78.17 (71.43–84.91)	89.26 (84.29–94.23)
	Hibou-B	87.02 (83.18–90.86)	76.96 (71.16–82.76)	72.77 (65.79–79.75)	84.14 (78.23–90.06)
	CONCH	87.18 (83.35–91.00)	79.98 (74.81–85.15)	77.98 (71.63–84.34)	83.28 (77.51–89.04)
	**UniCAS**	**91.70 (88.51–94.90)**	**84.72 (80.31–89.13)**	81.46 (75.52–87.40)	**89.79 (85.09–94.49)**
Clue Cell Diagnosis	Gigapath	97.74 (96.74–98.73)	93.56 (91.68–95.44)	94.30 (91.88–96.71)	92.44 (89.91–94.97)
	H-optimus	97.30 (96.26–98.34)	92.84 (90.91–94.78)	**94.44 (92.05–96.83)**	91.08 (88.09–94.08)
	UNI	97.64 (96.65–98.63)	93.02 (91.08–94.95)	93.53 (90.96–96.10)	92.28 (89.47–95.10)
	Hibou-B	97.48 (96.29–98.68)	94.18 (92.46–95.90)	93.96 (91.45–96.47)	94.58 (92.27–96.90)
	CONCH	96.90 (95.81–97.99)	91.62 (89.52–93.73)	91.40 (88.43–94.38)	92.08 (89.29–94.86)
	**UniCAS**	**97.81 (96.73–98.89)**	**94.24 (92.52–95.96)**	93.84 (91.28–96.39)	**94.61 (92.31–96.91)**

Table S3. Comparison of slide-level diagnosis results using DTFree (%, 95% CI). Related to Figure 2.

Task	Encoder	AUC	F1-score	Sensitivity	Specificity
Cancer Screening	Gigapath	92.00 (91.36–92.64)	85.17 (84.17–86.17)	82.00 (80.57–83.44)	89.44 (88.30–90.59)
	H-optimus	91.64 (90.93–92.36)	83.52 (82.44–84.59)	**83.52 (82.19–84.86)**	83.32 (81.89–84.74)
	UNI	90.50 (89.73–91.28)	82.38 (81.28–83.47)	80.20 (78.73–81.68)	85.62 (84.28–86.97)
	Hibou-B	91.02 (90.26–91.79)	83.02 (81.96–84.08)	81.51 (80.06–82.95)	85.10 (83.75–86.45)
	CONCH	88.71 (87.83–89.59)	80.82 (79.69–81.94)	81.39 (80.01–82.78)	80.14 (78.73–81.55)
	**UniCAS**	**92.33 (91.61–93.05)**	**86.24 (85.30–87.17)**	83.24 (81.86–84.63)	**90.20 (89.08–91.31)**
Candidiasis Testing	Gigapath	90.76 (87.50–94.02)	83.72 (79.18–88.27)	**83.92 (78.39–89.44)**	83.84 (78.12–89.57)
	H-optimus	90.80 (87.59–94.02)	82.50 (77.86–87.13)	80.49 (74.61–86.36)	85.08 (79.31–90.85)
	UNI	91.02 (87.71–94.32)	82.20 (77.49–86.92)	73.70 (66.89–80.50)	**95.22 (91.82–98.63)**
	Hibou-B	89.54 (85.99–93.10)	81.12 (75.99–86.25)	75.25 (68.32–82.17)	89.84 (84.93–94.74)
	CONCH	88.56 (84.73–92.38)	80.58 (75.71–85.44)	79.20 (73.05–85.34)	82.07 (75.68–88.46)
	**UniCAS**	**92.26 (89.41–95.12)**	**85.67 (81.48–89.86)**	81.86 (76.14–87.59)	90.50 (85.99–95.00)
Clue Cell Diagnosis	Gigapath	97.74 (96.72–98.76)	93.37 (91.42–95.32)	92.73 (90.06–95.41)	94.11 (91.67–96.55)
	H-optimus	97.64 (96.67–98.61)	93.37 (91.49–95.25)	92.52 (89.83–95.22)	94.19 (91.78–96.60)
	UNI	98.12 (97.27–98.97)	**93.90 (92.06–95.75)**	92.12 (89.43–94.81)	**95.79 (93.80–97.78)**
	Hibou-B	97.86 (96.83–98.88)	93.82 (92.04–95.59)	92.78 (90.08–95.49)	95.02 (92.66–97.37)
	CONCH	97.38 (96.40–98.37)	91.48 (89.53–93.42)	92.11 (89.36–94.86)	90.46 (87.50–93.43)
	**UniCAS**	**98.20 (97.31–99.10)**	93.84 (91.94–95.73)	**93.41 (90.81–96.01)**	94.51 (92.26–96.76)

Table S4. Class-wise metrics for slide-level fine-grained subtype diagnosis (%, 95%CI). Related to Figure 2.

Class	Encoder	Sensitivity	AUC	F1-score	Specificity
NILM	Gigapath	42.31 (26.47–58.14)	80.05 (72.42–87.69)	51.23 (35.80–66.67)	**94.57 (91.08–98.05)**
	H-optimus	72.35 (58.33–86.37)	83.22 (75.87–90.57)	59.55 (47.91–71.19)	82.47 (76.62–88.31)
	UNI	64.07 (48.65–79.49)	81.47 (73.59–89.36)	53.08 (40.68–65.49)	81.05 (75.15–86.96)
	Hibou-B	55.80 (40.47–71.12)	80.65 (73.26–88.05)	55.69 (43.08–68.30)	89.24 (84.35–94.12)
	CONCH	35.56 (20.00–51.11)	71.67 (63.34–80.00)	35.77 (21.54–50.00)	85.19 (79.58–90.80)
	**UniCAS**	**74.46 (61.11–87.81)**	**90.32 (86.16–94.49)**	**67.46 (56.41–78.51)**	88.32 (83.43–93.20)
ASC-US	Gigapath	22.19 (8.82–35.56)	**65.42 (55.58–75.26)**	24.54 (10.71–38.36)	85.60 (79.87–91.33)
	H-optimus	20.61 (7.89–33.33)	62.23 (53.45–71.01)	23.83 (10.17–37.50)	**88.13 (83.11–93.16)**
	UNI	18.85 (7.14–30.56)	61.91 (52.35–71.46)	21.23 (8.70–33.77)	86.08 (80.64–91.52)
	Hibou-B	**33.27 (18.92–47.62)**	61.84 (52.46–71.22)	**32.48 (19.17–45.78)**	82.89 (77.24–88.54)
	CONCH	18.09 (6.38–29.79)	48.68 (39.19–58.17)	17.49 (6.06–28.92)	78.40 (71.85–84.94)
	**UniCAS**	28.60 (14.71–42.50)	61.35 (51.92–70.78)	31.29 (17.24–45.33)	86.23 (80.79–91.67)
LSIL	Gigapath	**54.68 (38.76–70.59)**	72.85 (64.99–80.71)	**43.06 (30.76–55.36)**	74.95 (68.15–81.76)
	H-optimus	40.41 (25.00–55.81)	73.19 (65.65–80.73)	37.17 (24.32–50.01)	81.12 (75.00–87.25)
	UNI	43.45 (28.56–58.34)	71.38 (63.73–79.03)	40.75 (28.17–53.34)	82.94 (76.92–88.96)
	Hibou-B	38.02 (23.40–52.64)	71.99 (63.36–80.62)	38.28 (25.00–51.55)	**84.82 (79.22–90.41)**
	CONCH	38.25 (22.22–54.29)	73.38 (65.91–80.85)	35.21 (21.88–48.55)	81.09 (75.31–86.88)
	**UniCAS**	45.04 (30.56–59.52)	**73.87 (66.06–81.67)**	41.83 (28.92–54.74)	82.39 (76.47–88.31)
ASC-H	Gigapath	**45.13 (29.73–60.53)**	**71.05 (62.53–79.57)**	39.26 (26.67–51.86)	77.78 (71.52–84.05)
	H-optimus	25.21 (12.19–38.24)	69.55 (60.54–78.56)	26.90 (14.29–39.51)	84.71 (78.85–90.57)
	UNI	33.11 (18.60–47.62)	70.06 (59.75–80.38)	33.29 (20.00–46.58)	84.34 (78.81–89.86)
	Hibou-B	32.89 (18.42–47.37)	58.74 (47.50–69.97)	30.63 (17.50–43.75)	79.60 (73.29–85.90)
	CONCH	36.47 (21.43–51.52)	65.96 (56.59–75.34)	35.92 (22.49–49.35)	83.70 (77.92–89.48)
	**UniCAS**	40.87 (25.64–56.11)	66.24 (55.73–76.75)	**41.43 (27.78–55.07)**	**86.60 (81.17–92.03)**
HSIL	Gigapath	36.47 (20.00–52.95)	79.32 (70.88–87.76)	39.78 (24.00–55.56)	**91.07 (86.67–95.48)**
	H-optimus	**50.45 (33.33–67.57)**	79.77 (70.80–88.74)	**48.72 (34.28–63.16)**	88.84 (84.17–93.51)
	UNI	47.51 (30.30–64.71)	**81.79 (74.07–89.50)**	48.46 (33.96–62.96)	90.53 (86.06–95.00)
	Hibou-B	44.98 (28.20–61.76)	75.96 (67.43–84.48)	43.31 (29.03–57.58)	87.36 (82.17–92.55)
	CONCH	35.23 (19.35–51.11)	74.43 (65.07–83.80)	33.67 (19.44–47.89)	85.68 (80.11–91.25)
	**UniCAS**	46.87 (30.76–62.97)	80.98 (72.89–89.08)	46.74 (32.14–61.34)	89.13 (84.18–94.08)
Avg.	Gigapath	40.14 (33.36–46.91)	73.93 (69.52–78.34)	40.15 (33.16–47.15)	84.99 (83.25–86.73)
	H-optimus	41.43 (35.09–47.78)	73.80 (69.45–78.15)	39.48 (32.97–45.98)	85.26 (83.54–86.98)
	UNI	41.03 (34.37–47.70)	73.79 (69.61–77.97)	39.70 (32.92–46.49)	85.23 (83.54–86.92)
	Hibou-B	40.55 (33.78–47.31)	70.28 (65.56–75.00)	40.33 (33.89–46.78)	85.11 (83.45–86.77)
	CONCH	32.09 (25.52–38.66)	66.89 (62.49–71.29)	31.69 (25.22–38.16)	82.95 (81.29–84.62)
	**UniCAS**	**47.21 (40.73–53.69)**	**74.63 (70.18–79.09)**	**46.13 (39.80–52.46)**	**86.77 (85.11–88.42)**

Table S5. Comparison of region-level diagnoses for cervical abnormality classification (%, 95% CI). Related to Figure 3.

Encoder	ACC (Macro)	F1-score (Macro)	Sensitivity	Specificity
Gigapath	93.40 (93.14–93.67)	91.15 (90.80–91.50)	97.82 (97.51–98.13)	88.98 (88.57–89.38)
H-optimus	93.24 (92.99–93.50)	89.95 (89.58–90.32)	**99.00 (98.79–99.20)**	87.47 (87.02–87.92)
UNI	92.86 (92.59–93.13)	89.46 (89.09–89.84)	98.79 (98.56–99.02)	86.91 (86.43–87.39)
Hibou-B	92.72 (92.43–93.00)	89.76 (89.38–90.15)	97.41 (97.10–97.72)	88.00 (87.54–88.47)
CONCH	86.53 (86.22–86.85)	80.61 (80.14–81.08)	97.03 (96.82–97.24)	76.03 (75.39–76.64)
**UniCAS**	**94.23 (94.00–94.46)**	**91.47 (91.13–91.81)**	98.84 (98.62–99.07)	**89.63 (89.20–90.06)**

Table S6. Comparison of region-level diagnoses for candidiasis classification (%, 95% CI). Related to Figure 3.

Encoder	ACC	F1-score	Sensitivity	Specificity
Gigapath	95.53 (94.72–96.34)	95.45 (94.37–96.53)	92.55 (88.80–96.30)	**98.48 (96.96–100.0)**
H-optimus	95.62 (94.95–96.29)	95.59 (94.83–96.35)	94.42 (91.86–96.98)	96.83 (94.63–99.03)
UNI	95.82 (95.44–96.20)	95.84 (95.22–96.46)	94.76 (91.22–98.30)	96.78 (93.57–100.0)
Hibou-B	95.69 (95.08–96.30)	95.51 (94.85–96.17)	94.38 (91.55–97.21)	97.00 (94.55–99.45)
CONCH	93.79 (92.85–94.73)	93.64 (92.83–94.45)	92.05 (89.00–95.10)	95.62 (91.92–99.32)
**UniCAS**	**96.15 (95.82–96.48)**	**96.30 (95.86–96.74)**	**95.68 (94.25–97.11)**	96.62 (95.63–97.61)

Table S7. Comparison of few-shot experiments on clue cell classification (F1-score%, 95% CI). Related to Figure 3.

Encoder	K=1	K=2	K=4	K=8	K=16
Gigapath	**74.5 (71.9–77.1)**	78.6 (76.0–81.2)	86.1 (83.9–88.0)	95.2 (94.0–96.6)	97.3 (96.2–98.3)
H-optimus	66.6 (63.6–69.6)	66.3 (63.4–69.2)	89.8 (88.0–91.7)	92.5 (90.7–94.0)	96.7 (95.5–97.7)
UNI	68.1 (65.2–71.1)	67.2 (64.1–70.3)	85.9 (83.8–88.0)	85.1 (82.8–87.6)	96.4 (95.2–97.5)
Hibou-B	**74.5 (71.9–77.3)**	80.7 (78.3–83.0)	87.1 (85.2–89.2)	94.0 (93.0–95.0)	94.6 (93.2–96.0)
CONCH	50.1 (46.8–53.1)	60.0 (56.7–63.1)	68.1 (65.0–71.0)	73.3 (70.5–76.0)	84.3 (82.0–86.5)
**UniCAS**	**74.5 (71.8–77.1)**	**84.3 (81.8–86.6)**	**91.7 (90.0–93.4)**	**96.7 (95.5–97.7)**	**98.4 (97.6–99.1)**

Table S8. Region-level cervical abnormality grading results (95% CI). Related to Figure 3.

Encoder	Top-1 ACC	Top-2 ACC	Sensitivity	F1-score
Gigapath	67.97 (67.36–68.58)	93.25 (92.86–93.64)	59.88 (59.18–60.58)	58.18 (56.79–59.57)
H-optimus	67.97 (67.01–68.93)	92.98 (92.21–93.75)	61.10 (59.58–62.62)	59.06 (55.56–62.56)
UNI	67.49 (66.76–68.22)	93.27 (92.89–93.65)	61.33 (60.94–61.72)	60.83 (60.42–61.24)
Hibou-B	66.37 (65.59–67.15)	92.09 (91.69–92.49)	61.95 (60.66–63.24)	62.31 (61.55–63.07)
CONCH	65.34 (64.75–65.93)	91.76 (91.46–92.06)	58.69 (58.14–59.24)	59.02 (58.35–59.69)
**UniCAS**	**70.25 (69.51–70.99)**	**94.93 (94.61–95.25)**	**69.71 (68.93–70.49)**	**67.08 (66.08–68.08)**

Table S9. Comparison of region-level abnormal cervical cell detection (95% CI). Related to Figure 4.

Encoder	AP	AP50	AP75	APm
Gigapath	28.65 (26.46–30.84)	58.49 (54.78–62.19)	25.23 (21.91–28.55)	31.91 (28.22–35.60)
H-optimus	15.35 (13.84–16.86)	42.09 (38.62–45.55)	7.25 (5.58–8.92)	13.59 (11.23–15.95)
UNI	26.65 (24.69–28.61)	58.37 (54.91–61.83)	21.81 (18.81–24.81)	30.25 (26.53–33.96)
Hibou-B	10.76 (9.42–12.09)	29.16 (25.87–32.44)	4.98 (3.67–6.29)	7.75 (5.55–9.95)
CONCH	25.55 (23.41–27.68)	52.06 (48.53–55.58)	22.28 (19.28–25.27)	29.12 (25.70–32.53)
**UniCAS**	**29.99 (27.80–32.17)**	**62.67 (59.32–66.01)**	**26.48 (23.13–29.82)**	**34.65 (30.74–38.55)**

Table S10. Comparison of region-level clue cell detection (95% CI). Related to Figure 4.

Encoder	AP	AP50	AP75	APm
Gigapath	27.80 (24.02–31.57)	56.74 (49.91–63.57)	25.10 (20.02–30.17)	28.44 (24.10–32.77)
H-optimus	20.01 (17.20–22.82)	51.96 (46.04–57.87)	8.91 (6.27–11.55)	17.02 (13.98–20.05)
UNI	26.40 (22.89–29.90)	56.77 (51.09–62.44)	20.78 (15.92–25.63)	27.55 (22.97–32.13)
Hibou-B	22.35 (19.34–25.35)	51.67 (45.36–57.97)	14.84 (10.98–18.70)	23.74 (19.96–27.52)
CONCH	26.95 (22.93–30.96)	55.67 (49.13–62.21)	22.10 (16.79–27.41)	29.48 (25.07–33.89)
**UniCAS**	**30.06 (26.23–33.88)**	**60.48 (54.43–66.52)**	**26.27 (20.74–31.79)**	**30.05 (25.83–34.26)**

Table S11. Comparison of region-level fine-grained subtype detection (AP, 95% CI). Related to Figure 4.

Encoder	ASC-US	LSIL	ASC-H	HSIL	Avg.
Gigapath	8.76 (6.96–10.55)	18.27 (15.27–21.26)	15.41 (12.91–17.90)	7.15 (4.98–9.31)	12.47 (11.41–13.52)
H-optimus	6.14 (4.62–7.66)	11.48 (9.20–13.76)	12.11 (9.67–14.55)	5.30 (3.31–7.29)	8.73 (7.81–9.65)
UNI	10.86 (8.49–13.22)	19.17 (16.06–22.27)	15.26 (12.50–18.02)	10.08 (6.52–13.64)	13.87 (12.49–15.24)
Hibou-B	2.27 (1.46–3.07)	4.73 (3.36–6.09)	8.59 (6.75–10.42)	1.59 (0.82–2.36)	4.23 (3.61–4.85)
CONCH	6.75 (5.16–8.33)	13.82 (11.36–16.27)	13.55 (10.90–16.20)	5.00 (3.25–6.74)	9.76 (8.87–10.65)
**UniCAS**	**12.40 (9.65–15.15)**	**21.06 (17.46–24.65)**	**17.87 (14.88–20.85)**	**14.75 (10.15–19.34)**	**16.62 (14.98–18.25)**

Table S12. Comparison of region-level fine-grained subtype detection (AP50, 95% CI). Related to Figure 4.

Encoder	ASC-US	LSIL	ASC-H	HSIL	Avg.
Gigapath	19.38 (15.06–23.69)	36.16 (30.72–41.60)	30.17 (25.92–34.42)	12.81 (9.08–16.54)	24.78 (22.81–26.74)
H-optimus	16.32 (12.43–20.20)	25.81 (21.40–30.22)	26.25 (21.56–30.93)	11.43 (7.52–15.34)	19.92 (18.11–21.73)
UNI	22.72 (18.14–27.30)	39.23 (33.74–44.71)	28.39 (23.87–32.90)	18.96 (13.18–24.74)	27.45 (25.13–29.77)
Hibou-B	6.89 (4.60–9.18)	14.17 (10.51–17.83)	20.18 (16.17–24.18)	4.28 (2.19–6.37)	11.19 (9.73–12.64)
CONCH	15.80 (12.54–19.06)	31.95 (26.81–37.08)	26.29 (21.64–30.94)	10.40 (7.25–13.54)	21.23 (19.64–22.81)
**UniCAS**	**24.73 (19.73–29.73)**	**42.54 (36.50–48.57)**	**30.57 (25.81–35.32)**	**26.56 (19.14–33.97)**	**31.43 (28.72–34.14)**

Table S13. Comparison of pixel-level cervical image enhancement (95% CI). Related to Figure 5.

Encoder	SSIM↑ (%)	PSNR↑	LPIPS↓
Gigapath	74.23 (60.22–88.24)	**26.92 (20.78–33.06)**	0.1340 (0.0764–0.1916)
H-optimus	72.92 (57.87–87.97)	26.59 (20.51–32.67)	0.1353 (0.0784–0.1922)
UNI	73.20 (58.37–88.03)	26.67 (20.38–32.96)	0.1392 (0.0816–0.1968)
Hibou-B	72.04 (56.41–87.67)	26.44 (20.19–32.69)	0.1401 (0.0802–0.2000)
CONCH	72.67 (57.48–87.86)	26.68 (20.38–32.98)	0.1343 (0.0780–0.1906)
**UniCAS**	**74.45 (60.30–88.60)**	**26.92 (20.66–33.18)**	**0.1332 (0.0762–0.1902)**

Table S14. External validation on the BMT dataset(%, 95% CI). Related to Figure 6.

Class	Encoder	Sensitivity	AUC	F1-score	Specificity
NILM	Gigapath	84.02 (73.17–94.87)	90.03 (85.02–95.03)	75.54 (65.93–85.15)	80.67 (72.15–89.19)
	H-optimus	60.83 (46.66–75.00)	88.74 (83.43–94.04)	62.04 (50.00–74.08)	84.04 (76.18–91.89)
	UNI	71.40 (57.50–85.29)	95.84 (92.71–98.96)	79.28 (69.08–89.47)	**96.00 (92.00–100.0)**
	Hibou-B	84.65 (74.18–95.12)	96.26 (93.72–98.81)	83.15 (74.63–91.67)	90.97 (84.88–97.06)
	CONCH	65.00 (50.00–80.00)	79.94 (71.47–88.42)	64.79 (52.50–77.08)	83.48 (75.29–91.67)
	**UniCAS**	**93.42 (86.84–100.0)**	**99.19 (98.37–100.0)**	**93.21 (87.72–98.70)**	95.83 (91.66–100.0)
LSIL	Gigapath	52.10 (36.11–68.09)	86.14 (79.69–92.59)	61.04 (46.67–75.41)	92.02 (86.48–97.56)
	H-optimus	53.37 (37.50–69.23)	83.35 (76.44–90.25)	54.07 (40.67–67.47)	79.67 (70.59–88.75)
	UNI	84.06 (73.68–94.44)	93.13 (88.83–97.43)	78.40 (69.13–87.67)	84.35 (76.19–92.50)
	Hibou-B	66.67 (51.28–82.05)	91.15 (86.32–95.98)	69.91 (58.33–81.48)	88.25 (81.33–95.18)
	CONCH	53.02 (38.30–67.74)	73.92 (64.74–83.09)	55.62 (42.42–68.82)	82.16 (74.07–90.24)
	**UniCAS**	**86.73 (76.08–97.37)**	**98.54 (97.24–99.85)**	**89.85 (82.86–96.84)**	**96.75 (93.51–100.0)**
HSIL	Gigapath	95.95 (91.89–100.0)	98.05 (96.12–99.97)	91.97 (86.07–97.87)	93.20 (87.67–98.73)
	H-optimus	88.55 (79.49–97.62)	96.18 (93.09–99.28)	82.89 (74.42–91.36)	87.30 (80.49–94.12)
	UNI	91.83 (83.66–100.0)	97.79 (95.86–99.73)	89.61 (82.67–96.55)	93.05 (87.36–98.73)
	Hibou-B	91.67 (83.33–100.0)	98.03 (96.43–99.62)	88.30 (80.95–95.65)	91.88 (86.11–97.65)
	CONCH	83.67 (72.34–95.00)	90.07 (84.56–95.58)	77.89 (68.40–87.38)	84.47 (76.54–92.41)
	**UniCAS**	**100.0 (100.0–100.0)**	**99.79 (99.59–100.0)**	**96.75 (93.51–100.0)**	**96.71 (93.42–100.0)**
Avg.	Gigapath	78.53 (71.96–85.09)	91.70 (87.94–95.45)	77.13 (69.69–84.58)	89.01 (85.33–92.68)
	H-optimus	67.79 (60.08–75.50)	89.48 (85.34–93.61)	66.94 (59.05–74.83)	83.70 (79.41–87.99)
	UNI	83.09 (76.47–89.71)	95.71 (93.13–98.29)	82.77 (76.04–89.50)	91.41 (88.04–94.78)
	Hibou-B	81.49 (74.88–88.10)	95.26 (92.67–97.84)	80.94 (74.10–87.77)	90.72 (87.27–94.17)
	CONCH	67.59 (59.88–75.29)	81.51 (75.73–87.29)	66.84 (58.82–74.87)	83.75 (79.62–87.88)
	**UniCAS**	**93.79 (89.60–97.98)**	**99.22 (98.51–99.92)**	**93.83 (89.54–98.11)**	**97.06 (94.99–99.12)**

Table S15. External validation of region-level classification (%, 95% CI). Related to Figure 6.

Dataset	Encoder	ACC	F1-score	Sensitivity	Specificity
Herlev	Gigapath	96.20 (93.48–98.91)	94.70 (91.02–98.37)	**94.96 (91.40–98.53)**	**94.86 (91.10–98.61)**
	H-optimus	94.30 (90.76–97.83)	93.10 (89.03–97.18)	94.68 (91.27–98.08)	94.81 (91.44–98.18)
	UNI	94.30 (90.76–97.83)	92.28 (88.07–96.50)	91.00 (85.57–96.43)	90.55 (85.22–95.88)
	Hibou-B	93.20 (89.67–96.74)	91.32 (86.70–95.93)	91.89 (86.92–96.86)	91.92 (87.51–96.34)
	CONCH	90.49 (86.41–94.57)	86.88 (81.17–92.59)	85.10 (79.04–91.15)	85.12 (78.37–91.88)
	**UniCAS**	**97.02 (94.57–99.46)**	**96.02 (92.78–99.26)**	94.73 (90.57–98.89)	94.50 (90.19–98.81)
SIPaKMeD	Gigapath	95.68 (94.32–97.04)	95.74 (94.38–97.10)	96.06 (94.83–97.28)	98.95 (98.62–99.28)
	H-optimus	94.88 (93.33–96.42)	94.74 (93.26–96.23)	95.23 (93.92–96.54)	98.76 (98.40–99.11)
	UNI	96.24 (94.94–97.53)	96.20 (94.92–97.47)	96.38 (95.15–97.61)	99.08 (98.76–99.41)
	Hibou-B	93.89 (92.22–95.56)	93.80 (92.22–95.38)	94.19 (92.71–95.67)	98.48 (98.08–98.89)
	CONCH	95.06 (93.70–96.42)	94.95 (93.44–96.46)	95.23 (93.82–96.64)	98.75 (98.38–99.12)
	**UniCAS**	**97.72 (96.79–98.64)**	**97.73 (96.72–98.74)**	**97.80 (96.88–98.73)**	**99.43 (99.17–99.69)**

Table S16. External validation of region-level detection on ComparisonDetector dataset (%, 95% CI). Related to Figure 6.

Encoder	mAP	AP50	AP75	APm
Gigapath	24.18 (21.43–26.94)	49.20 (46.01–52.40)	20.86 (17.12–24.61)	18.57 (12.16–25.01)
H-optimus	8.97 (7.71–10.29)	21.45 (18.81–24.09)	6.52 (4.48–8.55)	3.74 (2.56–4.91)
UNI	25.40 (22.76–28.04)	50.65 (46.84–54.47)	**23.63 (19.43–27.83)**	**20.66 (15.33–25.99)**
Hibou-B	6.29 (5.06–7.53)	16.32 (13.60–19.04)	2.72 (1.75–3.69)	2.35 (1.35–3.44)
CONCH	13.43 (11.51–15.35)	30.06 (27.22–32.93)	9.42 (7.08–11.76)	10.60 (7.13–14.07)
**UniCAS**	**26.99 (23.47–30.51)**	**53.59 (49.42–57.77)**	23.13 (18.06–28.20)	14.74 (12.50–16.98)

Table S17. External validation of region-level segmentation (%, 95% CI). Related to Figure 6.

Dataset	Encoder	AP	Dice	F1-score
ISBI 2014	Gigapath	65.60 (65.45–65.75)	90.72 (90.58–90.86)	91.79 (91.44–92.14)
	H-optimus	40.02 (39.24–40.80)	85.47 (85.30–85.63)	78.58 (77.92–79.25)
	UNI	62.33 (61.63–63.02)	91.21 (91.07–91.35)	94.04 (93.49–94.59)
	Hibou-B	19.62 (19.15–20.08)	80.93 (80.71–81.14)	43.18 (42.73–43.63)
	CONCH	57.57 (57.35–57.78)	89.83 (89.67–89.99)	88.11 (87.88–88.34)
	**UniCAS**	**69.09 (68.41–69.77)**	**92.11 (91.97–92.24)**	**96.13 (95.82–96.43)**
CNSeg	Gigapath	55.34 (54.86–55.81)	90.41 (90.38–90.43)	82.15 (81.90–82.40)
	H-optimus	43.58 (42.42–44.74)	87.57 (87.40–87.73)	63.04 (61.98–64.09)
	UNI	54.63 (54.27–54.98)	90.29 (90.27–90.31)	82.13 (81.65–82.60)
	Hibou-B	13.25 (12.64–13.85)	81.13 (80.96–81.29)	37.87 (36.97–38.77)
	CONCH	53.42 (52.05–54.78)	90.09 (89.93–90.25)	82.42 (81.59–83.25)
	**UniCAS**	**58.38 (58.28–58.47)**	**90.69 (90.64–90.74)**	**86.26 (85.63–86.88)**
Cx22-Multi	Gigapath	75.70 (74.27–77.13)	94.34 (94.12–94.56)	95.25 (94.47–96.03)
	H-optimus	60.12 (57.72–62.52)	90.50 (90.14–90.85)	87.37 (86.17–88.56)
	UNI	70.13 (68.39–71.87)	94.21 (93.98–94.43)	93.26 (92.41–94.10)
	Hibou-B	64.20 (62.28–66.12)	91.24 (90.91–91.56)	89.84 (88.72–90.95)
	CONCH	**79.52 (67.55–91.48)**	93.81 (93.58–94.04)	92.87 (91.96–93.77)
	**UniCAS**	77.71 (76.53–78.88)	**94.38 (94.16–94.59)**	**96.15 (95.37–96.92)**

Table S18. External validation on ISBI 2014 dataset (%, 95% CI). Related to Figure 6.

Encoder	mAP		Dice		F1-score	
	Cytoplasm	Nuclei	Cytoplasm	Nuclei	Cytoplasm	Nuclei
Gigapath	65.74 (64.88–66.59)	65.46 (64.71–66.21)	91.32 (90.72–91.92)	90.11 (90.08–90.14)	85.05 (84.67–85.43)	98.54 (98.15–98.92)
H-optimus	49.08 (47.89–50.26)	30.94 (30.30–31.58)	88.11 (87.87–88.34)	82.83 (82.65–83.01)	84.99 (84.26–85.72)	72.19 (71.18–73.19)
UNI	62.22 (61.05–63.39)	62.43 (61.91–62.95)	91.95 (91.70–92.20)	90.47 (90.34–90.59)	90.16 (89.34–90.98)	97.92 (97.63–98.21)
Hibou-B	37.57 (36.70–38.43)	1.67 (1.50–1.84)	84.61 (84.41–84.81)	77.25 (76.89–77.61)	80.97 (80.16–81.78)	5.39 (5.05–5.72)
CONCH	53.05 (52.87–53.22)	62.31 (61.87–62.75)	89.73 (89.51–89.95)	89.93 (89.72–90.14)	79.44 (78.96–79.92)	96.78 (96.27–97.29)
**UniCAS**	**71.33 (70.18–72.48)**	**66.88 (66.42–67.35)**	**92.99 (92.76–93.23)**	**91.23 (91.12–91.33)**	**93.63 (93.14–94.11)**	**98.64 (98.38–98.90)**

Table S19. Ablation study of Top-m in multi-task aggregator (%, 95% CI). Related to STAR Methods.

Task	Parameter	ACC	AUC	Sensitivity	Specificity
Cancer Screening	m=800	84.48 (84.01–84.95)	90.80 (90.53–91.07)	81.03 (80.20–81.86)	87.08 (85.53–88.62)
	m=512	84.73 (84.44–85.03)	91.08 (90.99–91.17)	81.60 (80.73–82.47)	**87.86 (86.91–88.81)**
	m=256	86.18 (85.77–86.60)	**93.09 (93.03–93.15)**	85.51 (85.30–85.71)	86.86 (86.07–87.68)
	m=128	**86.21 (85.54–86.89)**	92.26 (91.97–92.55)	85.94 (84.89–86.98)	86.48 (84.57–88.39)
	m=64	84.25 (83.38–85.11)	92.58 (92.38–92.78)	**86.64 (85.57–87.71)**	81.85 (79.15–84.55)
Candidiasis Testing	m=800	86.21 (84.86–87.56)	92.09 (91.27–92.92)	81.85 (79.46–84.24)	**90.57 (88.36–92.47)**
	m=512	**87.14 (85.86–88.41)**	93.00 (92.00–93.99)	85.42 (81.36–89.49)	88.85 (84.31–93.39)
	m=256	86.96 (85.99–87.94)	**93.21 (92.50–93.92)**	85.71 (84.80–86.63)	88.21 (86.50–89.93)
	m=128	86.13 (85.93–86.32)	92.45 (92.27–92.63)	86.18 (83.68–88.67)	86.07 (83.23–88.91)
	m=64	84.64 (84.11–85.17)	92.75 (92.20–93.29)	**86.96 (84.48–89.43)**	82.33 (81.46–83.20)
Clue Cell Diagnosis	m=800	94.02 (93.75–94.30)	98.17 (97.81–98.52)	94.27 (92.36–96.21)	93.85 (92.50–95.20)
	m=512	94.25 (93.69–94.80)	98.40 (98.30–98.50)	94.63 (93.34–95.91)	93.87 (92.55–95.19)
	m=256	**95.37 (94.69–96.05)**	**98.93 (98.45–99.40)**	95.62 (94.79–96.46)	**95.11 (93.45–96.78)**
	m=128	95.13 (94.58–95.67)	98.46 (98.39–98.53)	95.93 (95.15–96.72)	94.32 (93.07–95.58)
	m=64	95.14 (94.26–96.01)	98.44 (98.38–98.50)	**96.40 (95.73–97.06)**	94.03 (92.93–95.13)

Table S20. Ablation study of the number of experts (s) in multi-task aggregator (%, 95% CI). Related to STAR Methods.

Task	Parameter	ACC	AUC	Sensitivity	Specificity
Cancer Screening	s=2	85.16 (84.02–86.30)	92.15 (91.58–92.73)	83.50 (83.22–83.78)	86.82 (84.69–88.94)
	s=4	85.58 (85.33–85.82)	92.44 (92.20–92.68)	84.58 (83.23–85.93)	86.57 (85.54–87.61)
	s=8	**86.18 (85.77–86.60)**	**93.09 (93.03–93.15)**	**85.51 (85.30–85.71)**	86.86 (86.07–87.68)
	s=12	85.94 (85.27–86.60)	92.79 (92.40–93.18)	85.45 (84.47–86.44)	86.42 (85.21–87.64)
	s=16	86.01 (85.25–86.76)	92.69 (92.44–92.95)	85.08 (84.22–85.94)	**86.93 (84.69–89.18)**
Candidiasis Testing	s=2	85.71 (84.09–87.34)	92.46 (91.81–93.10)	84.71 (82.90–86.53)	86.71 (84.32–89.11)
	s=4	86.71 (85.32–88.10)	92.88 (91.95–93.81)	86.29 (83.46–89.11)	87.17 (83.69–90.59)
	s=8	86.96 (85.99–87.94)	93.21 (92.50–93.92)	85.71 (84.80–86.63)	88.21 (86.50–89.93)
	s=12	87.02 (84.55–89.50)	93.17 (92.14–94.19)	85.90 (84.92–86.89)	88.14 (84.14–92.15)
	s=16	**88.12 (86.96–89.29)**	**93.39 (92.52–94.26)**	**87.14 (84.66–89.63)**	**89.11 (86.28–91.93)**
Clue Cell Diagnosis	s=2	94.77 (94.15–95.39)	98.54 (98.13–98.95)	94.77 (93.79–95.76)	94.76 (93.78–95.74)
	s=4	95.28 (94.21–96.34)	98.29 (97.60–98.98)	96.34 (94.52–98.17)	94.21 (93.31–95.11)
	s=8	95.37 (94.69–96.05)	**98.93 (98.45–99.40)**	95.62 (94.79–96.46)	**95.11 (93.45–96.78)**
	s=12	**95.39 (94.79–96.04)**	98.78 (98.11–99.45)	95.68 (95.44–95.91)	**95.11 (93.61–96.61)**
	s=16	95.17 (95.05–95.29)	**98.93 (98.77–99.09)**	**96.35 (96.03–96.67)**	93.99 (93.48–94.49)
