# Supplementary material for: UniCAS: A foundation model for cervical cytology screening
Source: Cell Rep Med. 2026 Jan 20;7(1):102570. doi: 10.1016/j.xcrm.2025.102570 (PMC12866161; doi:10.1016/j.xcrm.2025.102570)
Supplement: Document S2. Article plus supplemental information [file mmc4.pdf]

Cell Reports Medicine

Article

UniCAS: A foundation model for cervical cytology screening

Graphical abstract

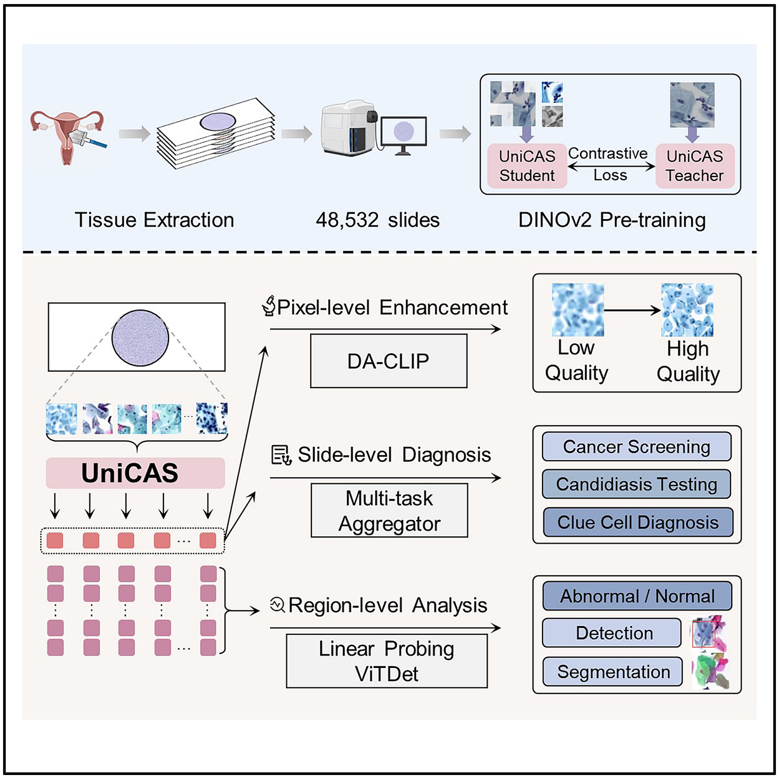

Highlights

- UniCAS is a cytology foundation model pre-trained on 48,532 cervical slides
- UniCAS outperforms other histopathology foundation models in various cytology tasks
- Multi-task aggregator concurrently handles slide-level tasks without performance drop
- A unified diagnostic pipeline can reduce the overall computational overhead by 70%

Authors

Haotian Jiang, Jiangdong Cai, Zhenrong Shen, ..., Dinggang Shen, Lichi Zhang, Qian Wang

Correspondence

lichizhang@sjtu.edu.cn (L.Z.),
qianwang@shanghaitech.edu.cn (Q.W.)

In brief

Jiang et al. present UniCAS, a foundation model pre-trained on 48,532 whole slide images for cervical cytology screening. By functioning as a unified encoder for multi-scale analysis, UniCAS enables various clinical analysis tasks with state-of-the-art performance, thereby addressing the inefficient and fragmented workflows of conventional approaches.

Jiang et al., 2026, Cell Reports Medicine 7, 102570
January 20, 2026 © 2025 The Authors. Published by Elsevier Inc.
https://doi.org/10.1016/j.xcrm.2025.102570

CellPress

Cell Reports Medicine

CellPress
OPEN ACCESS

Article

UniCAS: A foundation model for cervical cytology screening

Haotian Jiang,[1,7] Jiangdong Cai,[1,7] Zhenrong Shen,[2] Mengjie Xu,[1] Manman Fei,[2] Haolin Huang,[1] Xinyu Wang,[1] Rui Bi,[3,4] Dinggang Shen,[1,5,6] Lichi Zhang,[2,*] and Qian Wang[1,5,8,*]

[1]School of Biomedical Engineering & State Key Laboratory of Advanced Medical Materials and Devices, ShanghaiTech University, Shanghai, China

[2]School of Biomedical Engineering, Shanghai Jiao Tong University, Shanghai, China

[3]Department of Pathology, Fudan University Shanghai Cancer Center, Shanghai, China

[4]Department of Oncology, Shanghai Medical College, Fudan University, Shanghai, China

[5]Shanghai Clinical Research and Trial Center, Shanghai, China

[6]Shanghai United Imaging Intelligence Co. Ltd., Shanghai, China

[7]These authors contributed equally

[8]Lead contact

*Correspondence: lichizhang@sjtu.edu.cn (L.Z.), qianwang@shanghaitech.edu.cn (Q.W.)

https://doi.org/10.1016/j.xcrm.2025.102570

SUMMARY

Cervical abnormality screening is pivotal for prevention and treatment. However, the substantial size of whole slide images (WSIs) makes examination labor-intensive and time-consuming. Current deep learning-based approaches struggle with the morphological diversity of cervical cytology and require specialized models for distinct diagnostic tasks, leading to fragmented workflows. Here, we present UniCAS, a cytology foundation model pre-trained on 48,532 cervical WSIs encompassing diverse patient demographics and pathological conditions. UniCAS enables various clinical analysis tasks, achieving state-of-the-art performance in slide-level diagnosis, region-level analysis, and pixel-level image enhancement. In particular, by integrating a multi-task aggregator for slide-level diagnosis, UniCAS achieves area under the curve (AUC) values of 92.60%, 92.58%, and 98.39% for cancer screening, candidiasis testing, and clue cell diagnosis, respectively, while reducing diagnostic time by 70% compared with conventional approaches. This work establishes a paradigm for efficient multi-scale analysis in automated cervical cytology, bridging the gap between computational pathology and clinical diagnostic workflows.

INTRODUCTION

Cervical cancer remains a leading cause of cancer-related deaths in women worldwide, especially in low- and middle-income countries where healthcare resources are often limited.[1,2] Early detection through effective cervical screening is crucial for cancer prevention and the management of various pathological conditions. Among the screening methodologies, the ThinPrep Cytology Test (TCT) is recognized for its efficacy in identifying precancerous lesions, malignancies, and non-neoplastic conditions.[3–5] TCT involves meticulous collection and microscopic evaluation of cervical cells to detect abnormalities. This also includes the identification of specific conditions, such as candidiasis and the presence of clue cells.

Despite its benefits, the manual examination of TCT slides poses substantial challenges. This process is highly labor-intensive, time-consuming, and susceptible to variability among pathologists, which can undermine diagnostic accuracy. Furthermore, a global shortage of pathologists exacerbates these issues, highlighting an urgent need for efficient, automated systems to enhance diagnostic throughput and consistency.[6] Addressing these challenges is crucial to fully realize the benefits of TCT screening and enhance women's health worldwide, a process now significantly accelerated by advancements in artificial intelligence (AI).[7,8]

Deep learning-based automated cervical abnormality screening (CAS) systems offer the potential to greatly enhance diagnostic accuracy and streamline workflow efficiency.[9–12] Despite these prospects, the development of AI models is often hindered by the substantial requirement for extensive annotated datasets, which remains a challenging endeavor across varied clinical environments.[13–15] Moreover, deploying multiple task-specific models can complicate the diagnostic process, leading to inefficiencies and bottlenecks within clinical workflows.[16,17] Recently, advancements in foundation models have introduced a powerful paradigm through self-supervised learning on large datasets, showcasing promising generalization across a range of diagnostic tasks.[18–24] However, in computational pathology, these foundation models have predominantly been trained using histopathology data, which poses limitations to their applicability and effectiveness in cytology-centric tasks like CAS, primarily due to the significant morphological diversity present in these images.

© 2025 The Authors. Published by Elsevier Inc.
This is an open access article under the CC BY license (http://creativecommons.org/licenses/by/4.0/).

In this work, we advance the field of cervical cytology with the introduction of UniCAS, a vision transformer (ViT)-style model[25] specifically designed for identifying cervical abnormalities in TCT procedures. UniCAS is pre-trained on an exceptionally comprehensive dataset, comprising over 80 million image patches from 48,532 whole slide images (WSIs), representing individuals aged 15 to 90 (see Figures 1A and 1B; Table S1). The pre-training phase utilizes DINOv2,[26] a cutting-edge self-supervised learning method, executed over one million steps using these unlabeled data. This pre-training approach positions UniCAS to seamlessly integrate into various downstream cervical diagnostic tasks, functioning as a unified encoder for comprehensive analysis outlined in Figure 1C.

The practical effectiveness of UniCAS is rigorously assessed across multiple diagnostic tasks. At the slide level, UniCAS addresses complex challenges such as cancer screening, candidiasis testing, and clue cell diagnosis for individual cases. At the region level, UniCAS demonstrates capabilities in the classification, detection, and segmentation of cellular structures critical for accurate cervical cytology assessment on cropped regions from WSIs. UniCAS also tackles pixel-level enhancement to mitigate focus defects and image blurriness during TCT sample scanning. Overall, UniCAS demonstrates exceptional performance, consistently surpassing existing pathology foundation models (see Figure 1D).

Moreover, to streamline slide-level diagnosis, we introduce the multi-task aggregator. This innovation significantly reduces the diagnostic time required to perform all three slide-level tasks (cancer screening, candidiasis testing, and clue cell diagnosis) compared with conventional single-task methods (see Figure 1E). Building on this efficiency, we develop a fully automated diagnostic pipeline based on UniCAS that integrates this aggregator and other multi-scale analyses into a single, unified workflow. The pipeline begins with an optional pixel-level enhancement, followed by feature extraction using UniCAS. These shared features are then used for simultaneous slide-level diagnosis via the multi-task aggregator. For slides predicted as positive, the pipeline automatically proceeds to a detailed region-level analysis, such as abnormal cell detection, to localize suspicious areas. Finally, the system provides these consolidated results to pathologists, aiding them in the creation of the final screening report. By eliminating the redundant, sequential processing found in traditional frameworks, our unified pipeline reduces the overall computational overhead by 70%, marking a significant step toward practical clinical implementation (see Figure 1E).

RESULTS

In this study, we introduce UniCAS, a cytology foundation model specifically developed for TCT-based CAS. UniCAS demonstrates impressive capabilities across a range of diagnostic processes. Primarily, it significantly enhances slide-level diagnosis for TCT screening, improving both accuracy and efficiency. Furthermore, UniCAS expands its functionality to showcase comprehensive region-level diagnostic capabilities, which include regional classification, detection of abnormal cells, and segmentation of cell nuclei, solidifying its crucial role in interpretable cervical cytology diagnostics. In addition to these functionalities, UniCAS enhances imaging quality at the pixel level. This is especially beneficial for refining the visualization of WSIs, which may be corrupted during acquisition, leading to clearer imaging that better equips healthcare professionals to make precise diagnoses.

Boosting slide-level diagnosis

UniCAS is meticulously designed to optimize slide-level diagnostics, specifically focusing on three critical tasks: cancer screening, candidiasis testing, and clue cell diagnosis. This is achieved through a sophisticated process in which individual patches from WSIs are encoded and subsequently aggregated to attain robust slide-level classification. To effectively manage the aggregation of patch encodings from UniCAS, we employ two representative MIL aggregators, TransMIL[27] and DTFree,[28] both of which are shown to seamlessly incorporate the encodings. As illustrated in Figures 2A–2F, UniCAS significantly outperforms existing foundation models across all slide-level diagnostic tasks, underscoring its exceptional capabilities with these two aggregation techniques. Specifically, UniCAS achieves an area under the curve (AUC) of 90.90% with TransMIL and 92.33% with DTFree for cervical cancer screening. For candidiasis testing, it achieves an AUC of 91.70% with TransMIL and 92.26% with DTFree, while for clue cell diagnosis, it attains remarkable AUCs of 97.81% and 98.20% using TransMIL and DTFree, respectively. Notably, DTFree generally outperforms TransMIL across various experimental conditions, particularly when utilizing different foundation encoders under consideration.

Furthermore, emphasizing the interconnected nature of clinical diagnostic tasks, we explored UniCAS's potential within a multi-task framework. Unlike TransMIL and DTFree, which require separate training of aggregators for different tasks, we propose the multi-task aggregator to effectively replace them, thereby streamlining the diagnostic process into a single forward dataflow for each WSI. In comparative analyses, UniCAS outperforms existing foundation models with the proposed multi-task aggregator, achieving AUC scores of 92.60% for cancer screening, 92.58% for candidiasis testing, and 98.39% for clue cell diagnosis (Figures 2G–2I). Notably, in addition to streamlining the process, the multi-task aggregator achieves the highest AUC scores across all three tasks, surpassing the single-task results. Details regarding slide-level diagnoses are provided in Tables S2 and S3 and Data S1. To further assess the clinical utility for decision-making, we performed a decision curve analysis (Figure S1), in which UniCAS consistently provides a superior or comparable net benefit across all three diagnostic tasks relative to other foundation models.

Beyond the primary binary cancer screening task (positive or negative), we evaluate UniCAS's capability for fine-grained subtype diagnosis. In the five-class subtype diagnosis task, UniCAS achieves AUC scores of 90.32% for NILM, 61.35% for ASC-US, 73.87% for LSIL, 66.24% for ASC-H, and 80.98% for HSIL, resulting in an overall AUC of 74.63% across all subtypes. Detailed results for this multi-class diagnostic analysis are provided in Table S4.

CellPress
OPEN ACCESS

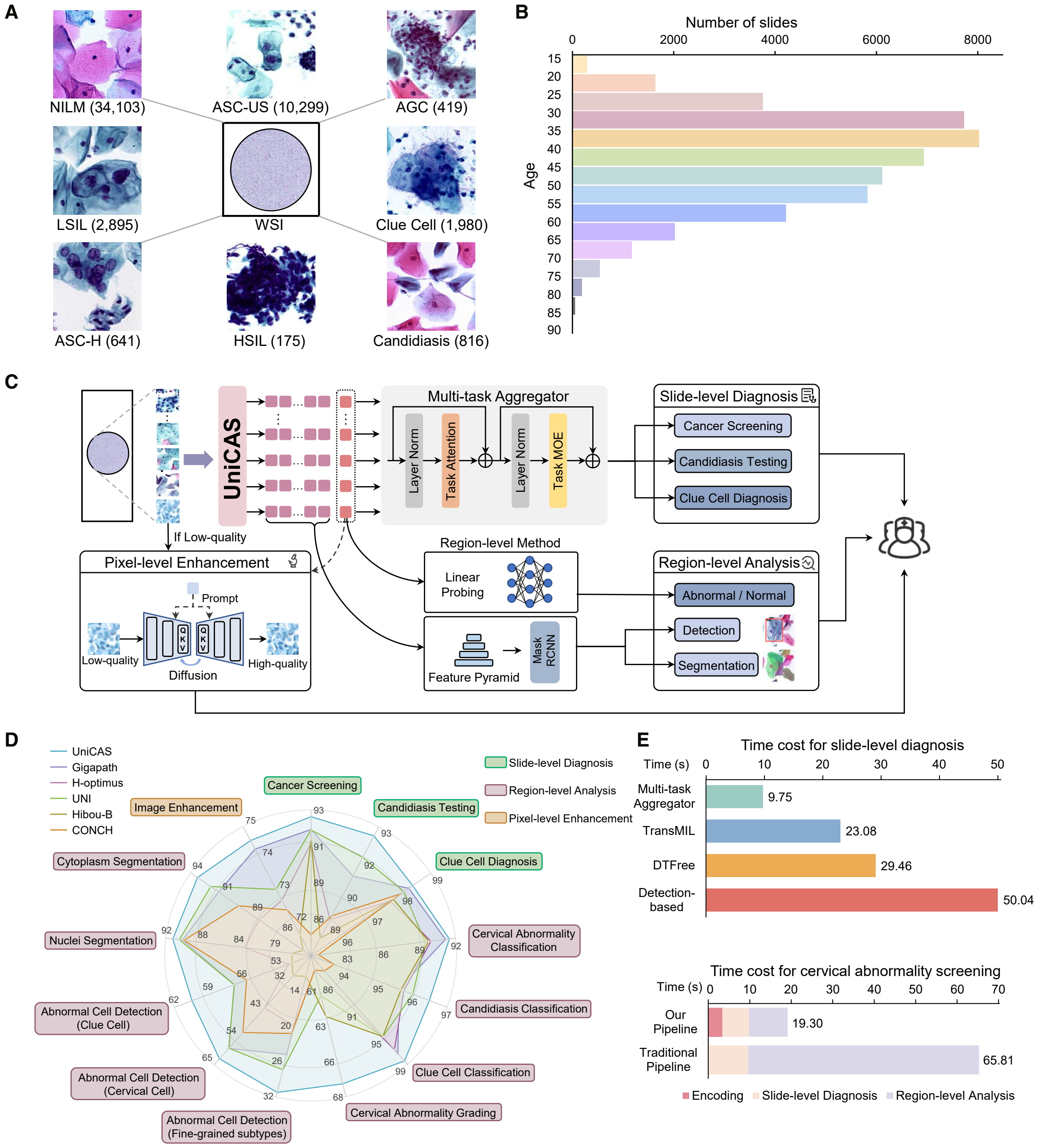

Figure 1. Overview of UniCAS, a foundation model pre-trained on cervical cytology data, which achieves state-of-the-art performance in cervical abnormality screening

(A) Training data distribution comprising 48,532 WSIs across various cervical abnormalities and infections.

(B) Age distribution of the pre-training dataset, ranging from 15 to 90 years.

(C) Diagnostic pipeline leveraging UniCAS, which seamlessly integrates slide-level diagnosis, region-level analysis, and pixel-level enhancement, enabling comprehensive cervical cytology evaluation.

(D) Performance comparison in cervical abnormality screening, showing that UniCAS consistently outperforms existing foundation models.

(E) Time efficiency comparison of our method against conventional approaches. The multi-task aggregator achieves up to 3× faster slide-level diagnosis compared with conventional approaches, and the full pipeline completes screening workflows at 3× the speed of the traditional pipeline.

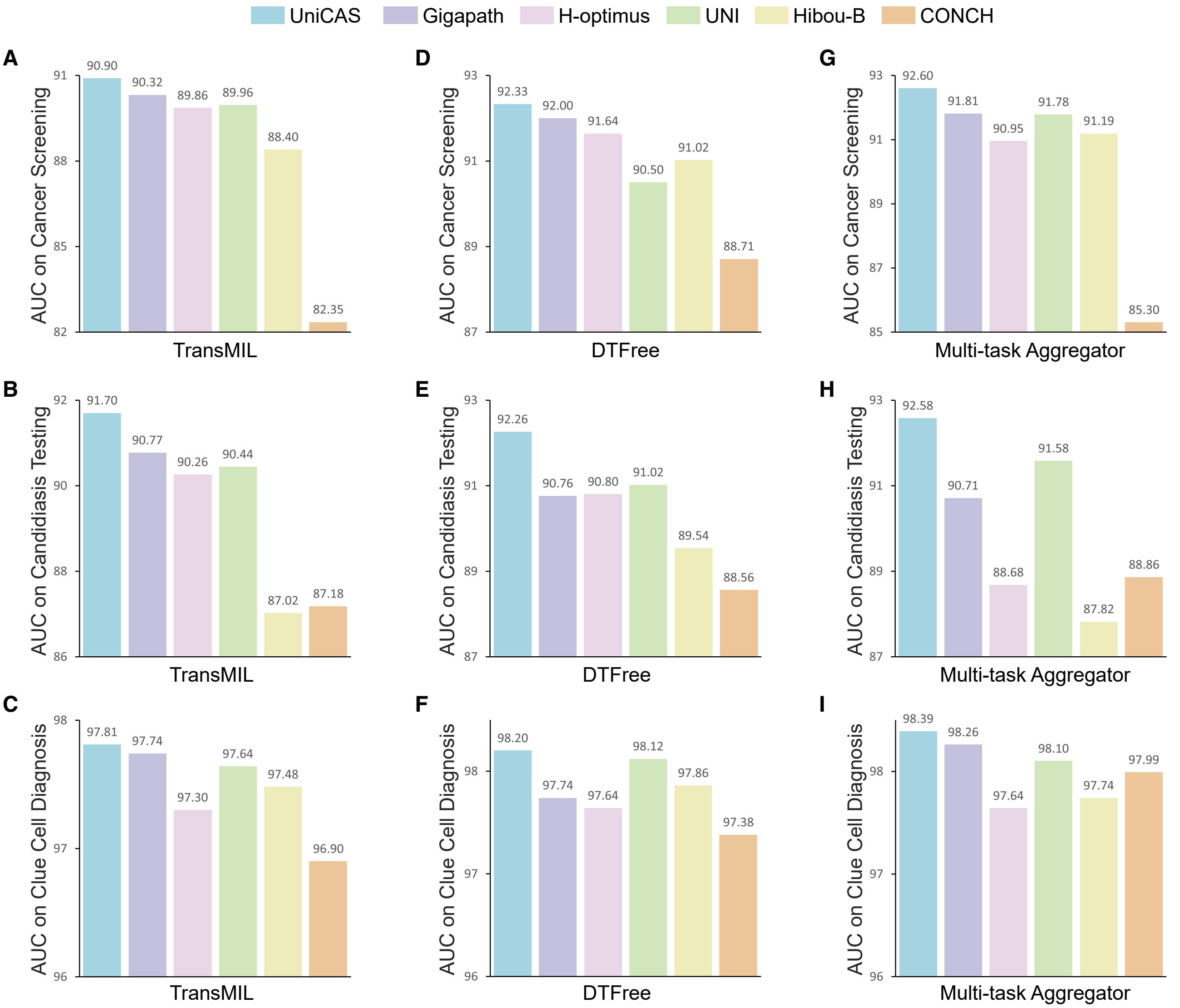

Figure 2. Slide-level diagnosis for three tasks: Cervical cancer screening, candidiasis testing, and clue cell diagnosis

(A)–(C) use TransMIL as the aggregator, while (D)–(F) use DTFree. (G)–(I) use our multi-task aggregator. Notably, both TransMIL and DTFree are designed for single tasks and trained individually for each diagnostic task, whereas the multi-task aggregator enables concurrent processing of all three tasks.

Additionally, the implementation of the multi-task aggregator facilitates the concurrent diagnosis of multiple tasks at the slide level, considerably enhancing processing efficiency. Our multi-task aggregator finishes slide-level screening in just 9.75 s per WSI, offering comparable diagnostic performance to conventional methods. This represents a significant improvement over traditional single-task approaches such as TransMIL (23.08 s), DTFree (29.46 s), and a detection-based method[29] (50.04 s). The efficiency gain allows our method to complete diagnoses in merely one-third of the time required by conventional techniques (Figure 1E). This advancement not only reduces diagnostic time but also highlights the practical utility of UniCAS in high-throughput clinical settings, where both speed and accuracy are critical.

Supporting region-level analysis

Region-level classification

UniCAS's region-level classification capabilities are highly significant in cervical cytology diagnostics. Through rigorous quantitative benchmarking using linear probing, UniCAS showcases superior discriminative power, achieving impressive F1 scores of 91.47% for cervical abnormality classification, 96.30% for candidiasis classification, and 98.44% for clue cell classification (Figures 3A–3C). Class activation mapping (CAM)[30] analysis further highlights its precise focus on diagnostically critical areas (Figure 3D). For example, UniCAS consistently focuses on nuclear abnormalities that are indicative of premalignant or malignant changes, such as irregular nuclear contours and chromatin hyperaggregation. In contrast, other foundation models often

CellPress
OPEN ACCESS

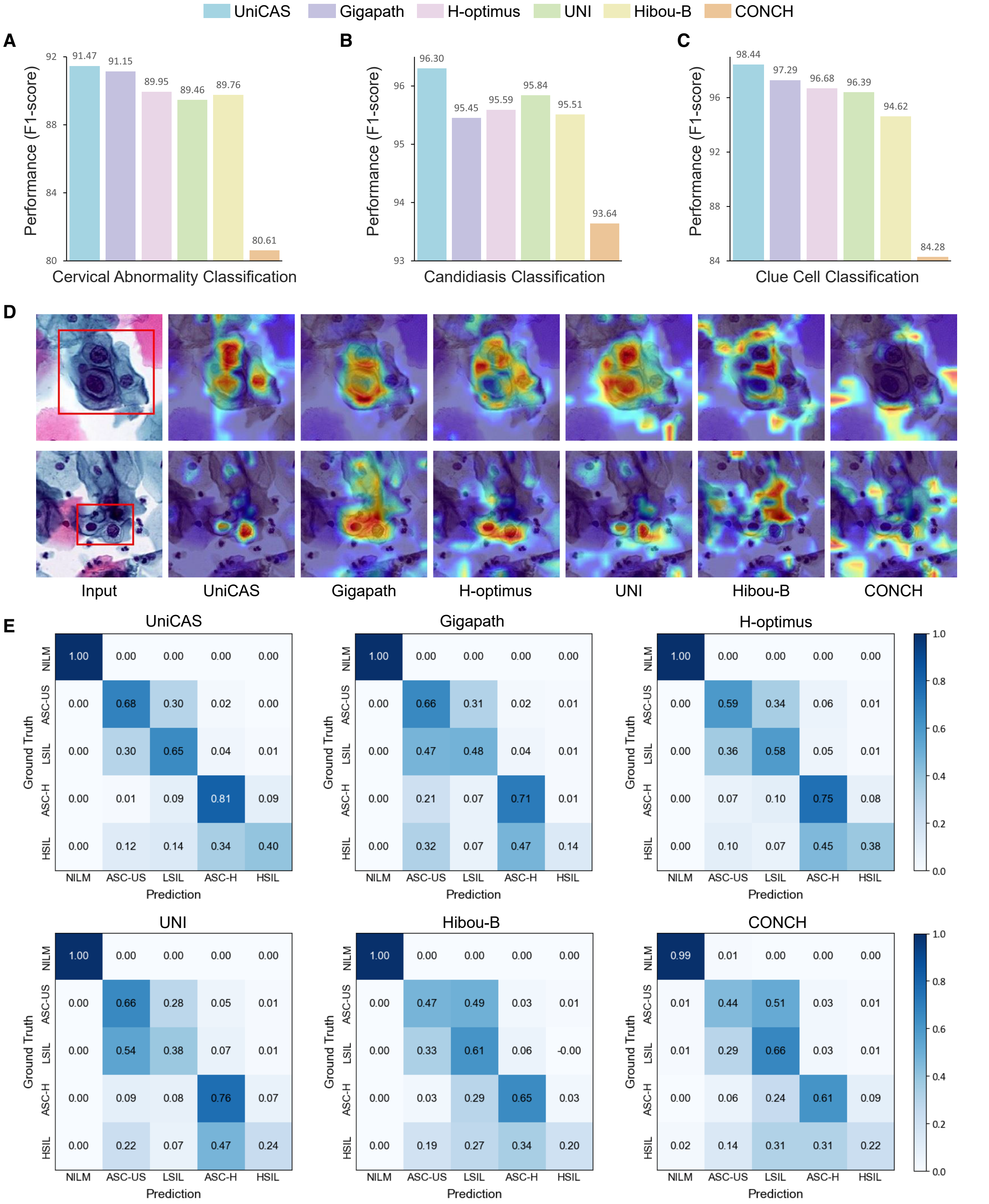

(legend on next page)

exhibit diffuse attention patterns, disproportionately emphasizing cytoplasmic areas.

Cervical abnormality grading

Additionally, we evaluate the performance of UniCAS in cervical abnormality grading at the region level, extending beyond binary classification to precisely categorize cervical squamous epithelial cells into five distinct classes: NILM, ASC-US, LSIL, ASC-H, and HSIL. UniCAS demonstrates superior accuracy, achieving 67.53% for ASC-US, 65.21% for LSIL, 81.40% for ASC-H, and 40.67% for HSIL, significantly outperforming other models (Figure 3E). In contrast, other foundation models typically exhibit strong discrimination abilities for negative cells (NILM) but often struggle with precise differentiation among specific grades of cervical abnormality. More details on region-level classification and grading are provided in Tables S5–S8. These results highlight UniCAS's robust performance in classification across various grades of cervical abnormality, particularly in comparison with other foundation models.

Abnormal cell detection

In the field of cervical screening, accurate abnormal cell detection is crucial for providing nuanced diagnostic insights beyond slide-level analysis. To address this, we evaluate UniCAS in the context of region-level detection tasks, specifically focusing on abnormal cervical squamous epithelial cells and clue cells (Figures 4A and 4B). For this evaluation, we integrate the ViTDet framework[31] with various ViT-style foundation models, including UniCAS, enabling direct comparison of their detection capabilities. UniCAS demonstrates remarkable performance, achieving the highest average precision (AP) score[32] of 29.99 in abnormal cervical cell detection and an AP score of 30.06 in clue cell detection (Figure 4C). When evaluated using the AP50 metric, UniCAS achieves scores of 62.67 and 60.48 for these detection tasks, respectively (Figure 4D). These scores significantly outperform those of other foundation models, highlighting UniCAS's exceptional ability to precisely identify abnormal cells while simultaneously minimizing false positive detections.

We further evaluate UniCAS's ability to perform fine-grained detection of abnormal cervical squamous epithelial cells, distinguishing between specific grades of abnormality rather than grouping them into a single category. For this fine-grained subtype detection task, UniCAS outperforms the comparative foundation models, achieving AP scores of 12.40 for ASC-US, 21.06 for LSIL, 17.87 for ASC-H, and 14.75 for HSIL (Figure 4E). The mean AP (mAP), which offers an overall performance measure across all categories, is reported at 16.62. More experimental results are provided in Tables S9–S12. These findings underscore UniCAS's enhanced capability to accurately identify and discern various grades of abnormal cervical cells, reinforcing its value as a powerful tool in cervical abnormality screening.

Enabling pixel-level enhancement

During the slide preparation process, misalignments in the scanner can lead to blurriness within WSIs, thereby affecting diagnostic accuracy. To counter this issue, we focus on validating the supplementary role of UniCAS in pixel-level enhancement, aiming to correct these imaging distortions and improve the clarity of WSIs for more accurate pathological assessment (Figure 5A). For this evaluation, we employ the DA-CLIP framework[33] to compare the enhancement performance of UniCAS against other foundation models. UniCAS excels in pixel-level cervical image enhancement, achieving the highest SSIM and PSNR scores of 74.45% and 26.92 dB among the models compared (Figures 5B and 5C). Moreover, UniCAS achieves the lowest LPIPS score of 0.1332 (Figure 5D), indicating that the enhanced images are perceptually closest to the ground truth reference images, as judged by human visual assessment. More details are provided in Table S13.

Generalizing to external datasets

External validation of slide-level diagnostic performance

To rigorously evaluate UniCAS's generalization capability, we validate its performance on external multi-center datasets derived from institutions with varying slide preparation protocols and scanner specifications. These external datasets are collected independently from the pre-training and internal training data, reflecting real-world variability in staining patterns and imaging conditions encountered in clinical practice and ensuring an unbiased assessment. Without requiring fine-tuning, UniCAS demonstrates exceptional performance, achieving AUCs of 90.10% for cancer screening, 89.92% for candidiasis testing, and 98.11% for clue cell diagnosis (Figures 6A–6C and Data S2).

Furthermore, we assess its slide-level cancer grading capabilities on the public BMT dataset.[34] UniCAS achieves outstanding AUCs of 99.19% for NILM, 98.54% for LSIL, and 99.79% for HSIL, confirming its robust generalization for cervical abnormality grading (Figure 6D; Table S14). These results surpass existing pathology foundation models and confirm UniCAS's robustness across diverse clinical workflows.

Comprehensive region-level validation on public datasets

To demonstrate the robustness and generalizability of UniCAS, we conduct a comprehensive validation of its region-level capabilities on multiple, widely recognized public datasets. This rigorous evaluation spans classification, detection, and segmentation, confirming its state-of-the-art performance across diverse and challenging benchmarks.

For region-level classification, we validate UniCAS on the Herlev[35] and SIPaKMeD[36] datasets. It achieves an exceptional F1

Figure 3. Region-level classification of abnormal cells

(A–C) Region-level classification performance (F1 score) for cervical abnormality classification, candidiasis classification, and clue cell classification using linear probing.

(D) Attention visualization produced by different foundation models.

(E) Confusion-matrix comparison of classification accuracy between UniCAS and other foundation models for cervical abnormality grading (NILM, ASC-US, LSIL, ASC-H, and HSIL).

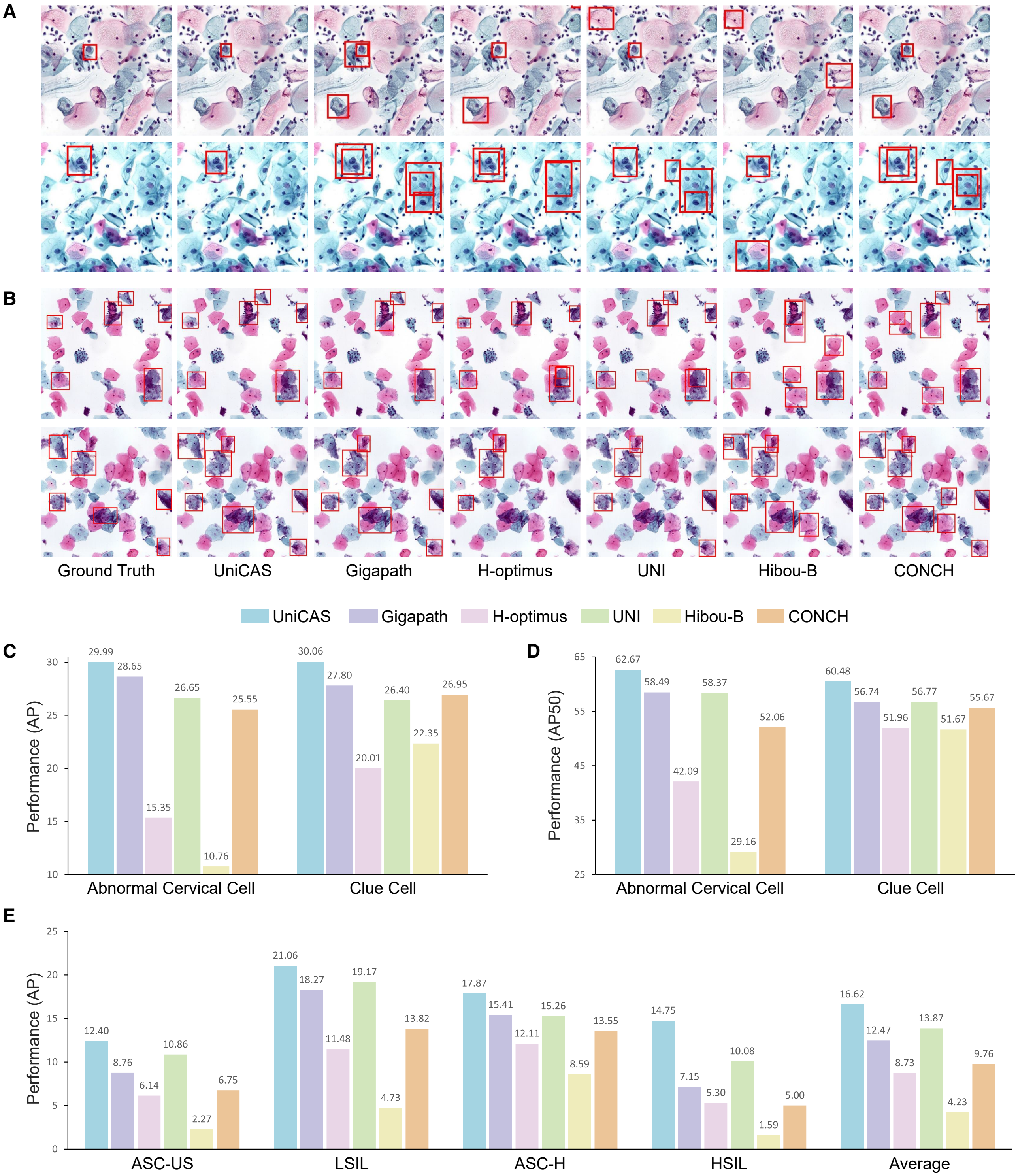

Figure 4. Region-level detection of abnormal cells

(A) Comparative detection results between UniCAS and other foundation models for abnormal cervical cells, showing that UniCAS effectively reduces the incidence of false-positive regions.

(B) Detection results for clue cells, where UniCAS demonstrates high sensitivity in clue cell localization.

Red boxes in (A) and (B) indicate annotated or model-detected abnormal cells.

(legend continued on next page)

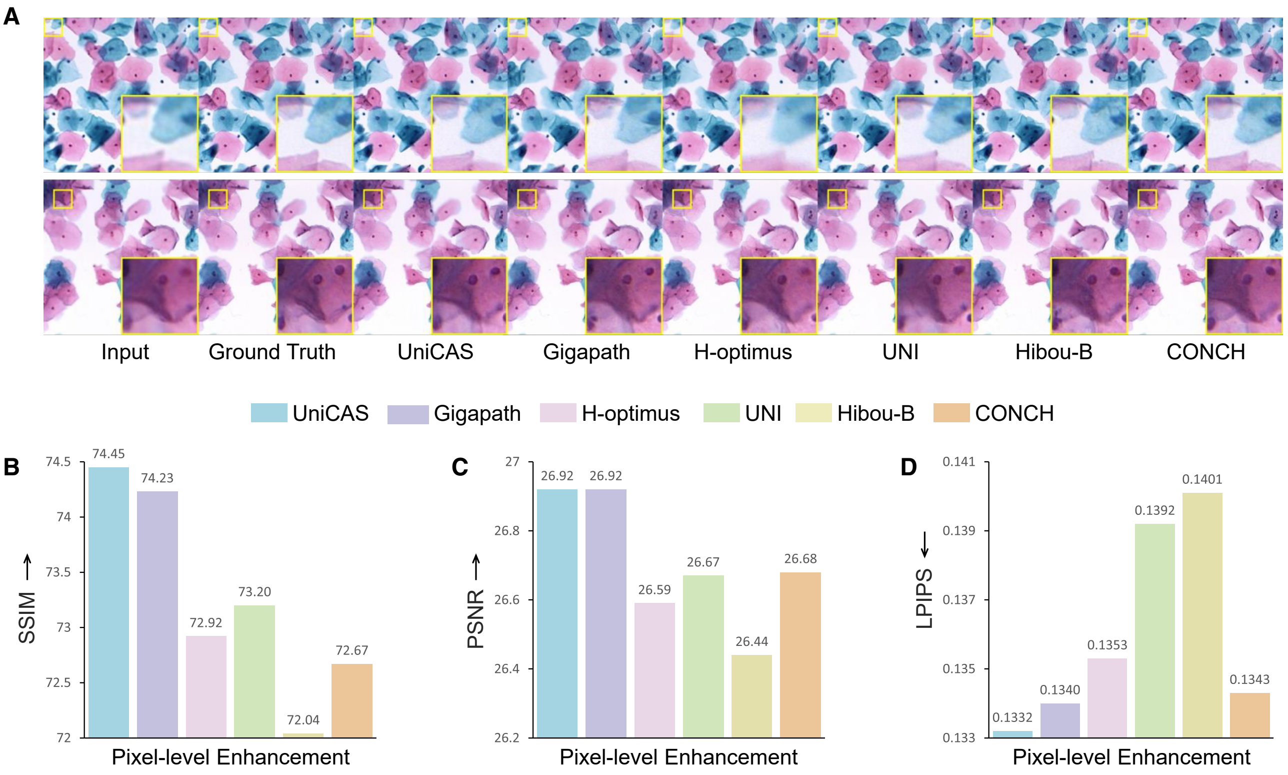

Figure 5. Pixel-level enhancement of blurred images

(A) Visual comparison of images enhanced by different foundation models. Regions in yellow bounding boxes are zoomed in for better visualization.

(B–D) Quantitative comparison using SSIM, PSNR, and LPIPS for pixel enhancement (↑: higher is better for SSIM/PSNR, ↓: lower is better for LPIPS).

score of 96.02% on Herlev and 97.73% on SIPaKMeD, showcasing its ability to accurately classify cellular abnormalities across datasets with different characteristics (Table S15). For region-level detection, we use the ComparisonDetector dataset,[37] where UniCAS achieves a leading mAP score of 26.99 (AP50 score of 53.59), proving its effectiveness in localizing abnormal cells in complex scenes (Table S16).

For region-level segmentation, a cornerstone for quantitative analysis such as nuclear-cytoplasmic ratio assessment, we validate UniCAS on three distinct public datasets: ISBI 2014,[38] CNSeg,[39] and Cx22-Multi.[40] On the ISBI 2014 challenge dataset, UniCAS excels at resolving overlapping cells (Figure 6E), achieving remarkable Dice scores of 92.99% for cytoplasm and 91.23% for nuclei, with an average of 92.11% (Figure 6F; Tables S17 and S18). Its strong performance is further confirmed on the CNSeg and Cx22-Multi datasets, where it achieves Dice scores of 90.69% and 94.38%, respectively. This consistent, high-level performance across multiple segmentation benchmarks validates UniCAS's clinical utility in providing the detailed structural insights essential for accurate cytological assessment.

DISCUSSION

This article introduces UniCAS, a unified foundation model specifically designed for diagnostic tasks associated with cervical abnormality screening. We compiled a comprehensive cervical TCT dataset comprising 48,532 WSIs, spanning a broad age range of 15–90 years and exhibiting diversity in staining and morphology. This dataset not only catalogs cervical cancer-related conditions, including NILM, ASC-US, LSIL, ASC-H, HSIL, and AGC, but also includes infections such as candidiasis and clue cells. The unprecedented scale and diversity of this dataset provide a robust foundation for model training, capturing the full spectrum of cellular variations and pathological manifestations encountered in clinical practice. Utilizing this extensive dataset, we have trained a ViT model using the self-supervised learning approach DINOv2,[26] which consistently outperforms other pathology foundation models, such as Gigapath,[18] H-optimus,[41] UNI,[20] Hibou-B,[21] and CONCH.[22]

UniCAS distinctly surpasses other foundation models across a wide range of cervical image tasks, yielding superior results in various assessments. At the slide level, UniCAS excels in cancer

(C) Region-level detection performance, measured by average precision (AP), for abnormal cervical cell and clue cell detection, comparing UniCAS with other foundation models using ViTDet.

(D) Region-level detection performance (AP50) of UniCAS and other foundation models for abnormal cervical cell and clue cell detection.

(E) AP scores for abnormal cell detection across fine-grained cervical abnormality subtypes.

CellPress
OPEN ACCESS

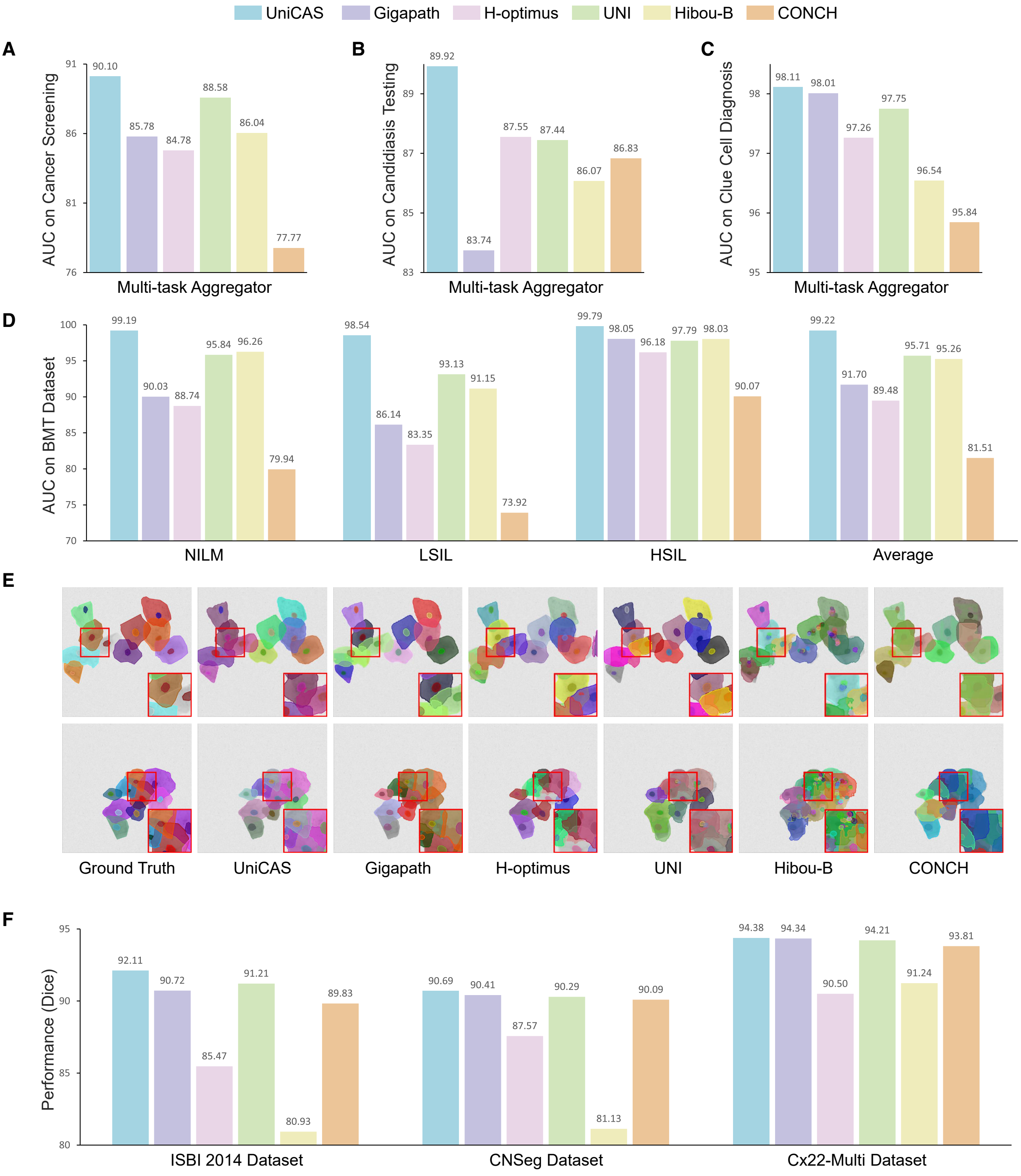

Figure 6. External validation of UniCAS and other foundation models

(A–C) Slide-level diagnosis performance (AUC) on cancer screening, candidiasis testing, and clue cell diagnosis using the multi-task aggregator, showing that UniCAS surpasses other foundation models in each task.

(D) Performance (AUC) on the BMT dataset, which consists of three classes: NILM, LSIL, and HSIL.

(E) Segmentation results of overlapping cells, with red bounding boxes highlighting regions with magnification for visual comparison. UniCAS demonstrates superior semantic understanding of cellular structures when segmenting overlapping cells.

(F) Region-level segmentation performance, assessed by Dice coefficient, evaluated on ISBI 2014, CNSeg, and Cx22-Multi datasets.

screening, candidiasis testing, and clue cell diagnosis, demonstrating robust diagnostic capability. At the region level, UniCAS effectively identifies abnormal cervical cells and infections while providing essential visual insights through the detection of these abnormalities and detailed segmentation of cellular structures. UniCAS also demonstrates superior performance in grading abnormal cervical cells, which improves clinical decision-making. Moreover, UniCAS introduces a powerful approach to address WSI blurriness through pixel-level enhancement capabilities, offering clearer and more consistent images than other pathology foundation models. This unprecedented improvement in image quality is crucial for delivering reliable diagnostic evidence, particularly in resource-constrained clinical settings where optimal scanning conditions may not be available.

Building upon UniCAS, we developed an intelligent diagnostic pipeline designed to ensure clinical trust through transparent and actionable results. To achieve this, the pipeline provides not only slide-level diagnosis but also detailed region-level visual evidence for suspicious regions. This includes bounding boxes around suspicious cells and highlights of high-confidence patches, allowing pathologists to quickly verify the model's findings and trace the evidence supporting the diagnosis. By processing WSIs with UniCAS and delivering a consolidated report containing both diagnostic results and this crucial visual feedback, the pipeline functions as a reliable aid that keeps the pathologist at the center of the workflow, ensuring safe and effective integration into clinical practice.

The significant reduction in processing time directly addresses a critical global healthcare challenge, as it enables high-throughput cervical cancer screening programs capable of serving large populations across diverse geographical regions.[42] The improved efficiency facilitates innovative care delivery approaches, such as centralized laboratory systems where patients can self-collect samples and mail them to processing centers capable of rapidly analyzing large volumes of specimens. Such centralized screening approaches are particularly valuable for low- and middle-income countries, where expanding screening coverage is essential for reducing cervical cancer mortality rates.[43] These remarkable efficiency gains can be attributed to two key technical innovations in our pipeline design. First, the multi-task aggregator eliminates redundant computations by sharing intermediate features across tasks, thereby reducing the overall computational load. Second, the unified framework minimizes the overhead associated with task-specific processing pipelines, allowing for more efficient resource utilization and faster diagnosis.

While recent advances in computational pathology have leveraged foundation models trained predominantly on histopathology data, these models face substantial limitations when applied to cytology-based cervical screening. Unlike histopathology, where tissue architecture and cellular relationships provide crucial diagnostic context, cervical cytology in TCT samples presents fundamentally different analytical challenges, with isolated cells dispersed across slides. These differences manifest in several key diagnostic aspects, as histopathology-based models struggle with the distinctive characteristics of cervical cytology, including the assessment of individual cell morphology, nuclear-cytoplasmic ratios, and chromatin patterns essential for accurate classification. Furthermore, cytology specimens exhibit considerable variability in preparation techniques, staining intensities, and cellular presentation compared with the relatively standardized histological sections, creating additional barriers to accurate computational analysis. UniCAS addresses these cytology-specific challenges through its pre-training on an extensive and diverse TCT dataset, enabling it to recognize subtle cellular abnormalities and structural patterns specific to cervical cytology. The robust performance advantage of UniCAS over histopathology-trained foundation models underscores the critical importance of domain-specific pre-training for computational cytology applications.

Limitations of the study

While UniCAS demonstrates superior performance across diverse cervical screening tasks through its domain-specific pre-training, several opportunities exist for further enhancing its clinical utility and applicability. First, although UniCAS effectively processes images at a fixed scale, expanding its capability to handle varying magnifications would improve its versatility in clinical settings, where examination at multiple magnification levels is common practice. This adaptation would enable pathologists to seamlessly analyze specimens across different optical resolutions without compromising diagnostic accuracy. Second, our study is limited by the absence of histopathological validation as a reference standard. The entire dataset originates from real-world clinical scenarios, containing only cytological data without corresponding histological gold-standard confirmation. Although inter-rater agreement analysis (Cohen's kappa) demonstrates reliable HSIL diagnosis among senior cytopathologists, the lack of histopathological follow-up for HSIL+ cases introduces potential diagnostic uncertainty. Third, integrating UniCAS with large language models represents a promising direction to improve interpretability and facilitate natural language interaction with the diagnostic system, as demonstrated by LLaVA-Med[44] and MetaGP.[45] Such multimodal integration would enable pathologists to query specific regions of interest, request explanations for diagnostic decisions, and generate comprehensive narrative reports that combine visual evidence with textual interpretation. Fourth, while our current implementation demonstrates robust performance in identifying various cervical abnormalities, extending the model to recognize additional rare pathological entities could further expand its diagnostic scope. Future work includes prospective multi-institutional studies to validate UniCAS's clinical impact and readiness for real-world adoption. In this way, UniCAS can be further recognized as a comprehensive solution for cervical cytology analysis while maintaining its computational efficiency and diagnostic accuracy. Moving forward, these avenues will be key to refining our methodology and improving the overall clinical impact of UniCAS on cervical screening programs worldwide.

RESOURCE AVAILABILITY

Lead contact

Requests for further information and resources should be directed to and will be fulfilled by the lead contact, Qian Wang (qianwang@shanghaitech.edu.cn).

Materials availability

This study did not generate new unique reagents.

Data and code availability

- All public datasets are listed under "deposited data" in the key resources table. The pre-training data used in this study are not publicly available due to patient privacy concerns and ethical restrictions. However, the region-level data reported in this paper can be provided upon reasonable request from the lead contact.
- Code and demo data for UniCAS are available at https://github.com/peter-fei/UniCAS.
- Any additional information required to reanalyze the data reported in this paper is available from the lead contact upon request.

ACKNOWLEDGMENTS

This work was supported in part by AI4S Initiative and HPC Platform of ShanghaiTech University and the National Natural Science Foundation of China (grant no. 62471288). The authors thank all members of the lab for their support.

AUTHOR CONTRIBUTIONS

Q.W., L.Z., and D.S. conceived, designed, and supervised the project. Q.W., L.Z., H.J., and J.C. created the cervical cytology datasets. H.J., J.C., and H.H. developed the pre-training of UniCAS. H.J., J.C., and M.F. developed the codebase for slide-level diagnosis. Z.S., M.X., H.H., and X.W. implemented the benchmarking codebase for pre-trained foundation models. R.B. provided medical consultation and guidance on cervical cytology. Q.W., H.J., and J.C. conducted the literature search and wrote the manuscript. All authors contributed to the review and editing of the manuscript.

DECLARATION OF INTERESTS

The authors have no conflicts of interest to declare.

STAR★METHODS

Detailed methods are provided in the online version of this paper and include the following:

- KEY RESOURCES TABLE
- EXPERIMENTAL MODEL AND STUDY PARTICIPANT DETAILS
  - Dataset for pre-training
  - Datasets for downstream tasks
- METHOD DETAILS
  - Pre-training of UniCAS encoder
  - Multi-task aggregator for slide-level diagnosis
  - Region-level analysis
  - Pixel-level enhancement
- QUANTIFICATION AND STATISTICAL ANALYSIS

SUPPLEMENTAL INFORMATION

Supplemental information can be found online at https://doi.org/10.1016/j.xcrm.2025.102570.

Received: June 17, 2025
Revised: September 17, 2025
Accepted: December 17, 2025
Published: January 20, 2026

REFERENCES

1. Bray, F., Laversanne, M., Sung, H., Ferlay, J., Siegel, R.L., Soerjomataram, I., and Jemal, A. (2024). Global cancer statistics 2022: Globocan estimates of incidence and mortality worldwide for 36 cancers in 185 countries. CA Cancer J. Clin. *74*, 229–263. https://doi.org/10.3322/caac.21834.
2. Siegel, R.L., Kratzer, T.B., Giaquinto, A.N., Sung, H., and Jemal, A. (2025). Cancer statistics, 2025. CA Cancer J. Clin. *75*, 10–45. https://doi.org/10.3322/caac.21871.
3. Monsonego, J., Autillo-Touati, A., Bergeron, C., Dachez, R., Liaras, J., Saurel, J., Zerat, L., Chatelain, P., and Mottot, C. (2001). Liquid-based cytology for primary cervical cancer screening: a multi-centre study. Br. J. Cancer *84*, 360–366. https://doi.org/10.1054/bjoc.2000.1588.
4. Abulafia, O., Pezzullo, J.C., and Sherer, D.M. (2003). Performance of ThinPrep liquid-based cervical cytology in comparison with conventionally prepared Papanicolaou smears: a quantitative survey. Gynecol. Oncol. *90*, 137–144. https://doi.org/10.1016/S0090-8258(03)00176-8.
5. Gibb, R.K., and Martens, M.G. (2011). The impact of liquid-based cytology in decreasing the incidence of cervical cancer. Rev. Obstet. Gynecol. *4*, S2–S11.
6. Bruni, L., Serrano, B., Roura, E., Alemany, L., Cowan, M., Herrero, R., Poljak, M., Murillo, R., Broutet, N., Riley, L.M., and de Sanjose, S. (2022). Cervical cancer screening programmes and age-specific coverage estimates for 202 countries and territories worldwide: a review and synthetic analysis. Lancet. Glob. Health *10*, e1115–e1127. https://doi.org/10.1016/S2214-109X(22)00241-8.
7. Van der Laak, J., Litjens, G., and Ciompi, F. (2021). Deep learning in histopathology: the path to the clinic. Nat. Med. *27*, 775–784. https://doi.org/10.1038/s41591-021-01343-4.
8. Jiang, H., Zhou, Y., Lin, Y., Chan, R.C.K., Liu, J., and Chen, H. (2023). Deep learning for computational cytology: A survey. Med. Image Anal. *84*, 102691. https://doi.org/10.1016/j.media.2022.102691.
9. Cheng, S., Liu, S., Yu, J., Rao, G., Xiao, Y., Han, W., Zhu, W., Lv, X., Li, N., Cai, J., et al. (2021). Robust whole slide image analysis for cervical cancer screening using deep learning. Nat. Commun. *12*, 5639. https://doi.org/10.1038/s41467-021-25296-x.
10. Zhu, X., Li, X., Ong, K., Zhang, W., Li, W., Li, L., Young, D., Su, Y., Shang, B., Peng, L., et al. (2021). Hybrid ai-assistive diagnostic model permits rapid tbs classification of cervical liquid-based thin-layer cell smears. Nat. Commun. *12*, 3541. https://doi.org/10.1038/s41467-021-23913-3.
11. Fei, M., Zhang, X., Cao, M., Shen, Z., Xiangyu, Z., Song, Z., Wang, Q., and Zhang, L. (2023). Robust cervical abnormal cell detection via distillation from local-scale consistency refinement. In Medical Image Computing and Computer Assisted Intervention – MICCAI 2023, H. Greenspan, A. Madabhushi, P. Mousavi, S. Salcudean, J. Duncan, T. Syeda-Mahmood, and R. Taylor, eds. (Springer), pp. 652–661. https://doi.org/10.1007/978-3-031-43987-2_63.
12. Cai, J., Xiong, H., Cao, M., Liu, L., Zhang, L., and Wang, Q. Progressive attention guidance for whole slide vulvovaginal candidiasis screening. In: Medical Image Computing and Computer Assisted Intervention – MICCAI 2023, H. Greenspan, A. Madabhushi, P. Mousavi, S. Salcudean, J. Duncan, T. Syeda-Mahmood, R. Taylor, eds. (Springer) https://doi.org/10.1007/978-3-031-43987-2_23.
13. Kermany, D.S., Goldbaum, M., Cai, W., Valentim, C.C.S., Liang, H., Baxter, S.L., McKeown, A., Yang, G., Wu, X., Yan, F., et al. (2018). Identifying medical diagnoses and treatable diseases by image-based deep learning. Cell *172*, 1122–1131.e9. https://doi.org/10.1016/j.cell.2018.02.010.
14. Bera, K., Schalper, K.A., Rimm, D.L., Velcheti, V., and Madabhushi, A. (2019). Artificial intelligence in digital pathology—new tools for diagnosis and precision oncology. Nat. Rev. Clin. Oncol. *16*, 703–715. https://doi.org/10.1038/s41571-019-0252-y.
15. Shmatko, A., Ghaffari Laleh, N., Gerstung, M., and Kather, J.N. (2022). Artificial intelligence in histopathology: enhancing cancer research and clinical oncology. Nat. Cancer *3*, 1026–1038. https://doi.org/10.1038/s43018-022-00436-4.

16. Cao, M., Zhang, X., Fan, X., Zhang, L., and Wang, Q. (2022). Parallel classification of cells in thinprep cytology test image for cervical cancer screening. In Cancer Prevention Through Early Detection, S. Ali, F. van der Sommen, B.W. Papież, M. van Eijnatten, Y. Jin, and I. Kolenbrander, eds. (Springer), pp. 34–43. https://doi.org/10.1007/978-3-031-17979-2_4.
17. Jiang, H., Zhang, R., Zhou, Y., Wang, Y., and Chen, H.D. (2023). Deep de-overlapping network for cytology instance segmentation. In 2023 IEEE/CVF Conference on Computer Vision and Pattern Recognition (CVPR) (IEEE), pp. 15641–15650. https://doi.org/10.1109/CVPR52729.2023.01501.
18. Xu, H., Usuyama, N., Bagga, J., Zhang, S., Rao, R., Naumann, T., Wong, C., Gero, Z., González, J., Gu, Y., et al. (2024). A whole-slide foundation model for digital pathology from real-world data. Nature *630*, 181–188. https://doi.org/10.1038/s41586-024-07441-w.
19. Tian, Y., Li, Z., Jin, Y., Wang, M., Wei, X., Zhao, L., Liu, Y., Liu, J., and Liu, C. (2024). Foundation model of ecg diagnosis: Diagnostics and explanations of any form and rhythm on ecg. Cell Rep. Med. *5*, 101875. https://doi.org/10.1016/j.xcrm.2024.101875.
20. Chen, R.J., Ding, T., Lu, M.Y., Williamson, D.F.K., Jaume, G., Song, A.H., Chen, B., Zhang, A., Shao, D., Shaban, M., et al. (2024). Towards a general-purpose foundation model for computational pathology. Nat. Med. *30*, 850–862. https://doi.org/10.1038/s41591-024-02857-3.
21. Nechaev, D., Pchelnikov, A., and Ivanova, E. (2024). Hibou: A family of foundational vision transformers for pathology. Preprint at arXiv. https://doi.org/10.48550/arXiv.2406.05074.
22. Lu, M.Y., Chen, B., Williamson, D.F.K., Chen, R.J., Liang, I., Ding, T., Jaume, G., Odintsov, I., Le, L.P., Gerber, G., et al. (2024). A visual-language foundation model for computational pathology. Nat. Med. *30*, 863–874. https://doi.org/10.1038/s41591-024-02856-4.
23. Peng, Y., Lin, A., Wang, M., Lin, T., Liu, L., Wu, J., Zou, K., Shi, T., Feng, L., Liang, Z., et al. (2025). Enhancing AI reliability: A foundation model with uncertainty estimation for optical coherence tomography-based retinal disease diagnosis. Cell Rep. Med. *6*, 101876. https://doi.org/10.1016/j.xcrm.2024.101876.
24. Wang, Y., Xiong, H., Sun, K., Bai, S., Dai, L., Ding, Z., Liu, J., Wang, Q., Liu, Q., and Shen, D. (2025). Toward general text-guided multimodal brain MRI synthesis for diagnosis and medical image analysis. Cell Rep. Med. *6*, 102182. https://doi.org/10.1016/j.xcrm.2025.102182.
25. Dosovitskiy, A., Beyer, L., Kolesnikov, A., Weissenborn, D., Zhai, X., Unterthiner, T., Dehghani, M., Minderer, M., Heigold, G., Gelly, S., et al. (2021). An image is worth 16x16 words: Transformers for image recognition at scale. In International Conference on Learning Representations.
26. Oquab, M., Darcet, T., Moutakanni, T., Vo, H., Szafraniec, M., Khalidov, V., Fernandez, P., Haziza, D., Massa, F., El-Nouby, A., et al. (2024). DINOv2: Learning robust visual features without supervision. Trans. Mach. Learn. Res.
27. Shao, Z., Bian, H., Chen, Y., Wang, Y., Zhang, J., Ji, X., and Zhang, Y. (2021). TransMIL: Transformer based correlated multiple instance learning for whole slide image classification. In Adv. Neural Inf. Process. Syst. (Curran Associates, Inc.), pp. 2136–2147.
28. Cao, M., Fei, M., Cai, J., Liu, L., Zhang, L., and Wang, Q. (2023). Detection-free pipeline for cervical cancer screening of whole slide images. In Medical Image Computing and Computer Assisted Intervention – MICCAI 2023, H. Greenspan, A. Madabhushi, P. Mousavi, S. Salcudean, J. Duncan, T. Syeda-Mahmood, and R. Taylor, eds. (Springer), pp. 243–252. https://doi.org/10.1007/978-3-031-43987-2_24.
29. Zhang, X., Cao, M., Wang, S., Sun, J., Fan, X., Wang, Q., and Zhang, L. (2022). Whole slide cervical cancer screening using graph attention network and supervised contrastive learning. In Medical Image Computing and Computer Assisted Intervention – MICCAI 2022, L. Wang, Q. Dou, P.T. Fletcher, S. Speidel, and S. Li, eds. (Springer), pp. 202–211. https://doi.org/10.1007/978-3-031-16434-7_20.
30. Zhou, B., Khosla, A., Lapedriza, A., Oliva, A., and Torralba, A. (2016). Learning deep features for discriminative localization. In IEEE Conference on Computer Vision and Pattern Recognition (CVPR) (IEEE), pp. 2921–2929. https://doi.org/10.1109/CVPR.2016.319.
31. Li, Y., Mao, H., Girshick, R., and He, K. (2022). Exploring plain vision transformer backbones for object detection. In Computer Vision – ECCV 2022, S. Avidan, G. Brostow, M. Cissé, G.M. Farinella, and T. Hassner, eds. (Springer), pp. 280–296. https://doi.org/10.1007/978-3-031-20077-9_17.
32. Everingham, M., Van Gool, L., Williams, C.K.I., Winn, J., and Zisserman, A. (2010). The pascal visual object classes (voc) challenge. Int. J. Comput. Vis. *88*, 303–338. https://doi.org/10.1007/s11263-009-0275-4.
33. Luo, Z., Gustafsson, F.K., Zhao, Z., Sjölund, J., and Schön, T.B. (2024). Controlling vision-language models for multi-task image restoration. In The Twelfth International Conference on Learning Representations.
34. Welch, E.C., Lu, C., Sung, C.J., Zhang, C., Tripathi, A., and Ou, J. (2024). Bmt: A cross-validated thinprep pap cervical cytology dataset for machine learning model training and validation. Sci. Data *11*, 1444. https://doi.org/10.1038/s41597-024-04328-3.
35. Jantzen, J., Norup, J., Dounias, G., and Bjerregaard, B. (2005). Pap-smear benchmark data for pattern classification. In Proc. NiSIS 2005, pp. 1–9.
36. Plissiti, M.E., Dimitrakopoulos, P., Sfikas, G., Nikou, C., Krikoni, O., and Charchanti, A. (2018). Sipakmed: A new dataset for feature and image based classification of normal and pathological cervical cells in pap smear images. In 2018 25th IEEE International Conference on Image Processing (ICIP) (IEEE), pp. 3144–3148. https://doi.org/10.1109/ICIP.2018.8451588.
37. Liang, Y., Tang, Z., Yan, M., Chen, J., Liu, Q., and Xiang, Y. (2021). Comparison detector for cervical cell/clumps detection in the limited data scenario. Neurocomputing *437*, 195–205. https://doi.org/10.1016/j.neucom.2021.01.006.
38. Lu, Z., Carneiro, G., and Bradley, A.P. (2015). An improved joint optimization of multiple level set functions for the segmentation of overlapping cervical cells. IEEE Trans. Image Process. *24*, 1261–1272. https://doi.org/10.1109/TIP.2015.2389619.
39. Zhao, J., He, Y.J., Zhou, S.H., Qin, J., and Xie, Y.N. (2023). Cnseg: A dataset for cervical nuclear segmentation. Comput. Methods Programs Biomed. *241*, 107732. https://doi.org/10.1016/j.cmpb.2023.107732.
40. Liu, G., Ding, Q., Luo, H., Sha, M., Li, X., and Ju, M. (2022). Cx22: A new publicly available dataset for deep learning-based segmentation of cervical cytology images. Comput. Biol. Med. *150*, 106194. https://doi.org/10.1016/j.compbiomed.2022.106194.
41. Saillard, C., Jenatton, R., Llinares-López, F., Mariet, Z., Cahané, D., Durand, E., and Vert, J.-P. (2024). H-optimus-0 (Bioptimus). https://github.com/bioptimus/releases/tree/main/models/h-optimus/v0.
42. Gupta, S., Palmer, C., Bik, E.M., Cardenas, J.P., Nuñez, H., Kraal, L., Bird, S.W., Bowers, J., Smith, A., Walton, N.A., et al. (2018). Self-sampling for human papillomavirus testing: Increased cervical cancer screening participation and incorporation in international screening programs. Front. Public Health *6*, 77. https://doi.org/10.3389/fpubh.2018.00077.
43. Kassa, R.N., Shifti, D.M., Alemu, K., and Omigbodun, A.O. (2024). Integration of cervical cancer screening into healthcare facilities in low- and middle-income countries: A scoping review. PLOS Glob. Public Health *4*, e0003183. https://doi.org/10.1371/journal.pgph.0003183.
44. Li, C., Wong, C., Zhang, S., Usuyama, N., Liu, H., Yang, J., Naumann, T., Poon, H., and Gao, J. (2023). Llava-med: Training a large language-and-vision assistant for biomedicine in one day. Adv. Neural Inf. Process. Syst. *36*, 28541–28564.
45. Liu, F., Zhou, H., Wang, K., Yu, Y., Gao, Y., Sun, Z., Liu, S., Sun, S., Zou, Z., Li, Z., et al. (2025). Metagp: A generative foundation model integrating electronic health records and multimodal imaging for addressing unmet clinical needs. Cell Rep. Med. *6*, 102056. https://doi.org/10.1016/j.xcrm.2025.102056.
46. Nayar, R., and Wilbur, D.C. (2015). The Bethesda System for Reporting Cervical Cytology: Definitions, Criteria, and Explanatory Notes (Springer). https://doi.org/10.1007/978-3-319-11074-5.
47. Sasieni, P., Castanon, A., and Cuzick, J. (2009). Effectiveness of cervical screening with age: population based case-control study of prospectively recorded data. BMJ *339*, b2968. https://doi.org/10.1136/bmj.b2968.

CellPress
OPEN ACCESS

48. World Health Organization. Global Strategy to Accelerate the Elimination of Cervical Cancer as a Public Health Problem. (2020). https://www.who.int/publications/i/item/9789240014107.

49. Arbyn, M., Weiderpass, E., Bruni, L., de Sanjosé, S., Saraiya, M., Ferlay, J., and Bray, F. (2020). Estimates of incidence and mortality of cervical cancer in 2018: a worldwide analysis. Lancet. Glob. Health *8*, e191–e203. https://doi.org/10.1016/S2214-109X(19)30482-6.

50. Lu, Z., Carneiro, G., Bradley, A.P., Ushizima, D., Nosrati, M.S., Bianchi, A.G.C., Carneiro, C.M., and Hamarneh, G. (2017). Evaluation of three algorithms for the segmentation of overlapping cervical cells. IEEE J. Biomed. Health Inform. *21*, 441–450. https://doi.org/10.1109/JBHI.2016.2519686.

51. Zhang, H., Meng, Y., Zhao, Y., Qiao, Y., Yang, X., Coupland, S.E., and Zheng, Y. (2022). Dtfd-mil: Double-tier feature distillation multiple instance learning for histopathology whole slide image classification. In 2022 IEEE/CVF Conference on Computer Vision and Pattern Recognition (CVPR) (IEEE), pp. 18780–18790. https://doi.org/10.1109/CVPR52688.2022.01824.

52. Cao, M., Fei, M., Xiong, H., Zhang, X., Fan, X., Zhang, L., and Wang, Q. (2024). Patch-to-sample reasoning for cervical cancer screening of whole slide image. IEEE Trans. Artif. Intell. *5*, 2779–2789. https://doi.org/10.1109/TAI.2023.3323637.

53. Yang, S., Wang, Y., and Chen, H. (2024). Mambamil: Enhancing long sequence modeling with sequence reordering in computational pathology. In Medical Image Computing and Computer Assisted Intervention – MICCAI 2024, M.G. Linguraru, Q. Dou, A. Feragen, S. Giannarou, B. Glocker, K. Lekadir, and J.A. Schnabel, eds. (Springer), pp. 296–306. https://doi.org/10.1007/978-3-031-72083-3_28.

54. Janowczyk, A., and Madabhushi, A. (2016). Deep learning for digital pathology image analysis: A comprehensive tutorial with selected use cases. J. Pathol. Inform. *7*, 29. https://doi.org/10.4103/2153-3539.186902.

55. Campanella, G., Hanna, M.G., Geneslaw, L., Miraflor, A., Werneck Krauss Silva, V., Busam, K.J., Brogi, E., Reuter, V.E., Klimstra, D.S., and Fuchs, T.J. (2019). Clinical-grade computational pathology using weakly supervised deep learning on whole slide images. Nat. Med. *25*, 1301–1309. https://doi.org/10.1038/s41591-019-0508-1.

56. Jacobs, R.A., Jordan, M.I., Nowlan, S.J., and Hinton, G.E. (1991). Adaptive mixtures of local experts. Neural Comput. *3*, 79–87. https://doi.org/10.1162/neco.1991.3.1.79.

57. Puigcerver, J., Riquelme Ruiz, C., Mustafa, B., and Houlsby, N. (2024). From sparse to soft mixtures of experts. The Twelfth International Conference on Learning Representations, 28435–28445.

58. He, K., Chen, X., Xie, S., Li, Y., Dollár, P., and Girshick, R. (2022). Masked autoencoders are scalable vision learners. In 2022 IEEE/CVF Conference on Computer Vision and Pattern Recognition (CVPR) (IEEE), pp. 15979–15988. https://doi.org/10.1109/CVPR52688.2022.01553.

59. He, K., Gkioxari, G., Dollar, P., and Girshick, R. (2017). Mask R-CNN. In 2017 IEEE International Conference on Computer Vision (ICCV) (IEEE), pp. 2980–2988. https://doi.org/10.1109/ICCV.2017.322.

STAR★METHODS

KEY RESOURCES TABLE

REAGENT or RESOURCE	SOURCE	IDENTIFIER
**Deposited data**		
BMT	Welch et al.[34]	https://doi.org/10.7303/syn55259257
Herlev	Jantzen et al.[35]	https://mde-lab.aegean.gr/index.php/downloads
SIPaKMeD	Plissiti et al.[36]	https://www.cs.uoi.gr/∼marina/sipakmed.html
ComparisonDetector	Liang et al.[37]	https://pan.baidu.com/s/1ynvNKBmf-s9GaUkTEUvTwA?pwd=fphm
ISBI 2014	Lu et al.[38]	https://cs.adelaide.edu.au/∼carneiro/isbi14_challenge
CNSeg	Zhao et al.[39]	https://www.kaggle.com/datasets/zhaojing0522/cervical-nucleus-segmentation
Cx22-Multi	Liu et al.[40]	https://github.com/LGQ330/Cx22?tab=readme-ov-file
**Software and algorithms**		
UniCAS	This paper	https://github.com/peter-fei/UniCAS
Python version 3.11	Python Software Foundation	https://www.python.org
PyTorch 2.0.1 + 11.8	PyTorch	https://pytorch.org
DINOv2	Oquab et al.[26]	https://github.com/facebookresearch/dinov2
ViTDet	Li et al.[31]	https://github.com/facebookresearch/detectron2
DA-CLIP	Luo et al.[33]	https://github.com/Algolzw/daclip-uir
**Other**		
NVIDIA Tesla A100 GPU	NVIDIA	https://www.nvidia.com/en-us/data-center/a100/

EXPERIMENTAL MODEL AND STUDY PARTICIPANT DETAILS

Dataset for pre-training

This study was approved by the ShanghaiTech University Ethics Committee (protocol number 202299-V1, approved on Jan 4, 2023). We have compiled a substantial collection of WSIs from third-party pathology diagnostic centers, with IRB approval, to train UniCAS. These data incorporate samples from diverse geographical regions and various scanning devices that exhibit differences in staining intensity and scanning protocols. In clinical practice, cytologists often refer to the Bethesda System (TBS) criteria for evaluating TCT samples.[46] To ensure high data quality, we rigorously curate the dataset by excluding cases with poor image quality, indistinct staining, insufficient cell concentrations, or other technical inadequacies. The refined dataset contains 48,532 samples in total, and each patient provides only one slide. This includes 34,103 normal cases (NILM) and 14,429 abnormal cases for cancer screening (comprising ASC-US, LSIL, ASC-H, AGC, and HSIL), as well as cases of infections (candidiasis and clue cells). Figure 1A illustrates the distribution of all these diagnostic categories, showing the specific counts for each category: ASC-US (10,299), LSIL (2,895), ASC-H (641), AGC (419), HSIL (175), candidiasis (816), and clue cells (1,980). The age distribution spans subjects from 15 to 90 years, with the majority concentrated in the 30 to 45 age group, which is considered a high-risk demographic for cervical diseases[47–49] (Figure 1B; Table S1).

To facilitate deep learning-based modeling, each WSI is cropped into 1,600 to 4,800 non-overlapping 256 × 256-pixel patches, collectively resulting in a total of 80,314,640 patches. All patches are extracted at 20× magnification, a resolution selected to preserve sufficient cellular detail for morphological analysis while ensuring computational efficiency during model training. These patches capture cellular images with diverse staining patterns (e.g., nuclear/cytoplasmic contrast variations) and morphological variations (including cell size and shape), thereby providing a rich dataset for cervical cytology research. UniCAS is trained to encode these diverse patches, learning robust visual representations that capture the subtle morphological features essential for accurate cervical abnormality screening.

Datasets for downstream tasks

To evaluate UniCAS, we create specialized datasets spanning three analytical levels: slide-level (for cancer screening, candidiasis testing, and clue cell diagnosis), region-level (for classification, grading, detection, and segmentation tasks), and pixel-level (for image enhancement). Each dataset addresses specific challenges in cervical screening. All datasets undergo meticulous annotation and validation by at least two experienced cytologists to ensure diagnostic accuracy.

Slide-level diagnosis

For slide-level diagnosis, we compile comprehensive datasets for three key diagnostic tasks: cancer screening, candidiasis testing, and clue cell diagnosis. These datasets consist primarily of WSIs from our pre-training collection, supplemented with additional data from the same pathology centers to evaluate model performance within familiar data distributions. All WSIs in these datasets carry slide-level diagnostic labels verified by experienced cytologists. Since UniCAS provides encodings for individual patches of each WSI, we aggregate these representations to generate slide-level features for classification. For all three tasks, we split the internal datasets into fixed training, validation, and test sets. We maintain class balance in each dataset to ensure unbiased evaluation and accurate assessment of model performance. The internal datasets are collected between May 2022 and November 2023 from 11 scanning devices, which inherently introduces variability in acquisition parameters and staining protocols. For external validation, we use 2,129 WSIs from six previously unseen scanning devices and 175 additional WSIs collected in January 2025, forming a comprehensive external test set of 2,304 WSIs. Each task's external validation is conducted using 1:1 balanced subsets derived from this combined dataset. The specific dataset compositions are detailed in the following sections.

Cancer screening. For cancer screening evaluation, we utilize two distinct datasets. The first internal dataset comprises 28,110 WSIs (14,055 positive and 14,055 negative) representing diverse demographic groups. Of these, 13,849 positive samples and 13,991 negative samples originate from our pre-training collection, while the remaining samples are acquired separately. We split this dataset into training (19,894), validation (2,210), and test (6,006) sets. The second dataset, used exclusively for external validation to assess generalization capability, includes 2,012 WSIs (1,006 positive and 1,006 negative) from an independent cohort not used in any aspect of model development or pre-training.

Candidiasis testing. The internal dataset contains 1,506 WSIs (753 positive and 753 negative). Of these, 570 positive and 728 negative samples originate from our pre-training collection, with the remaining 208 samples (183 positive and 25 negative) collected from supplementary sources. This dataset is partitioned into training, validation, and test sets using an 8:1:2 ratio, resulting in 1,054, 150, and 302 slides, respectively. Independent validation is performed on a separate dataset of 156 WSIs, containing 78 positive and 78 negative slides.

Clue cell diagnosis. The internal dataset includes 3,616 WSIs (1,808 positive and 1,808 negative), where all positive cases contain $\geq$20% clue cells per high-power field according to diagnostic guidelines. Among these, 1,275 positive samples and 1,736 negative samples are derived from pre-training data, with the remaining samples collected separately. For evaluation, the dataset is divided into a training set (2,530 slides), a validation set (360 slides), and a test set (726 slides) following an 8:1:2 distribution. The external validation dataset, used to assess generalization capability, contains 242 positive and 242 negative WSIs from independent sources.

Subtype diagnosis. The dataset for fine-grained subtype diagnosis comprises 970 WSIs spanning five distinct cytological classes: NILM (200), ASC-US (200), LSIL (200), ASC-H (200), and HSIL (170). This composition allows for the evaluation of multi-class diagnostic performance across the spectrum of cervical cytology. The WSIs within this dataset are collected using the same acquisition parameters (e.g., magnification and scanner sites) as those utilized for the slide-level tasks. For the comprehensive performance assessment, the dataset is subjected to 5-fold cross-validation, with the data partitioned into an approximately 4:1 ratio for training and testing in each fold.

Region-level classification

In addition to delivering slide-level diagnoses, UniCAS provides detailed diagnostic evidence at the region level. The data used for these classification tasks is entirely independent of the data used for pre-training UniCAS. For this purpose, positive patches containing abnormal cells or structures are meticulously selected by cytologists from regions of interest within positive WSIs. When creating the positive dataset, cytologists annotated not only the positive regions but also a small number of normal regions within the same WSIs. Therefore, negative patches are obtained from two sources: entire WSIs diagnosed as negative by clinicians, and normal regions within positive WSIs that do not contain diagnostic abnormalities. To mitigate potential bias, negative patches derived from positive WSIs are limited to less than 10% of the total negative dataset, with the vast majority sourced from definitively negative WSIs. Each region-level annotation undergoes rigorous validation by an additional cytologist after initial assessment, ensuring high accuracy and reliability throughout the diagnostic process.

Cervical abnormality classification. Curated from multiple hospitals between 2021 and 2023, this dataset contains 74,250 regions (37,125 normal and 37,125 abnormal) from 21,928 slides, alongside 12,372 hard negative regions from 2,859 negative slides. These hard negative regions consist of false positives generated by the detection model and are used exclusively for evaluation. All regions are 224×224 pixels at $20\times$ magnification, matching the global crop dimensions used during pre-training to enable direct utilization of patch encoding for classification. To ensure an unbiased evaluation, the dataset is partitioned at the patient level into stratified 4:1 train-test splits, and we apply 5-fold cross-validation.

Candidiasis classification. This dataset, collected by a collaborating clinical institute in 2021, consists of data carefully selected by cytologists. It includes 2,065 positive patches, 1,934 negative patches randomly cropped from negative WSIs, and 161 normal patches selected from positive WSIs. The dataset is divided at the patient level into stratified train-test sets in a 4:1 ratio, with 5-fold cross-validation applied to validate the results.

Clue cell classification. This dataset is designed for few-shot classification, where only 16 patches collected in 2021 are used for training. The evaluation is conducted on a separate test set of 2,000 patches (1,000 positive and 1,000 negative) collected between 2021 and 2023. Despite the limited training samples, this approach proves effective as clue cells exhibit distinctive morphological features that can be effectively captured by the pre-trained model.

Cervical abnormality grading. This dataset encompasses 5,970 patches collected between 2021 and 2023 across various grades: NILM (1,412), ASC-US (1,618), ASC-H (945), LSIL (1,680), and HSIL (315) within 224 $\times$ 224 pixels, and all NILM patches are from negative WSIs. To ensure a valid evaluation, the dataset is split at the patient level into a 4:1 train-test ratio and subjected to 5-fold cross-validation.

Region-level detection

Accurate cell detection enables the precise identification and characterization of pathological features within cervical cytology specimens. To evaluate the localization capabilities of UniCAS, we assemble dedicated detection datasets comprising abnormal cervical cells and clue cells with expert-annotated bounding boxes. These annotated images provide spatial information critical for guiding cytologists to regions of diagnostic interest within a slide. To prevent data leakage, the datasets for each detection task are partitioned at the patient level into training, validation, and test sets in an 8:1:1 ratio.

Cervical cell. This dataset contains 4,483 annotated regions (1024 $\times$ 1024 pixels) from 1,793 patients. For each WSI, no more than four regions are annotated by cytologists, resulting in 6,414 expert-annotated bounding boxes for abnormal cervical squamous epithelial cells. The annotations represent a comprehensive range of cellular abnormalities across the spectrum of cervical intraepithelial lesions. Each cellular annotation is precisely demarcated with a bounding box locating the abnormal cell within its contextual tissue environment.

Clue cell. This dataset, collected in 2021 from 181 patients, includes 510 regions (1024 $\times$ 1024 pixels) identified as positive for clue cells. Each positive region contains one or more clue cells, with a total of 3,533 individual instances meticulously annotated by cytologists.

Fine-grained subtypes. This dataset comprises 3,651 high-resolution regions of interest (1024 $\times$ 1024 pixels) extracted from ThinPrep cytology slides collected between 2021 and 2023 from 2,788 patients across multiple clinical sites. Expert cytologists manually annotated a total of 9,091 abnormal cells, applying strict Bethesda System morphological criteria. The annotations are subdivided into four diagnostically significant categories: ASC-US (2,364), LSIL (2,924), ASC-H (2,744), and HSIL (1,059).

Pixel-level enhancement

Beyond feature representation for diagnostic tasks, we explore UniCAS's capabilities in pixel-level image enhancement to address blurriness issues in cervical cytology WSIs. In an ideal case, WSIs are created by scanning through multiple focal planes and integrating them through sophisticated algorithms to generate a single sharp image. However, in routine settings, scanning multiple focal planes is often impractical due to time constraints, leading to single-plane scans that may be significantly off-focus and produce blurry images. Our enhancement task addresses this practical challenge by improving the quality of these potentially blurry single-plane images to match the clarity of optimally focused multi-plane integrated images.

To systematically evaluate this capability, we have assembled a specialized dataset comprising 550 WSIs from distinct patients. To create high-quality ground truth reference images, each slide is intentionally scanned through nine focal planes with equidistant spacing of 0.8 μm between adjacent planes. These focal planes are carefully calibrated to encompass the optimal focus range of each slide, ensuring that the integrated multi-plane images capture all relevant diagnostic details with maximum clarity. We randomly select 10 field-of-views from each WSI to train and evaluate the enhancement model, using out-of-focus planes as input and the integrated multi-plane images as ground truth. The slides and their selected views are allocated into training, validation, and test sets at an 8:1:1 ratio, encompassing cropped images from all focal planes.

Public dataset

To rigorously evaluate the versatility and performance of UniCAS, we benchmark it against a comprehensive suite of public datasets spanning multiple core tasks in computational cytology. These tasks include: slide-level classification (BMT[34] dataset), region-level classification (Herlev[35] and SIPaKMeD[36] datasets), region-level detection (ComparisonDetector[37] dataset), and region-level segmentation (ISBI 2014,[38] CNSeg,[39] and Cx22-Multi[40] datasets).

BMT. This publicly available multicellular ThinPrep dataset comprises 600 clinically vetted images collected from 180 Pap smear slides from 180 patients. The dataset is balanced and classified into three key diagnostic categories: NILM, LSIL, and HSIL, with 200 images per class. Each image has been independently analyzed and validated by three board-certified pathologists to ensure accurate classification.

Herlev. The Herlev dataset is a widely used public benchmark for cervical cell classification, containing 917 images of individual cervical cells from conventional Pap smear images. These images are categorized into seven distinct classes: Superficial squamous (74), Intermediate squamous (70), Columnar epithelial (98), Mild dysplasia (182), Moderate dysplasia (146), Severe dysplasia (197), and Carcinoma *in situ* (150). For simplified evaluation, these are grouped into normal class (for Superficial squamous, Intermediate squamous, Columnar epithelial) and abnormal class (for others).

SIPaKMeD. The SIPaKMeD dataset contains 4,049 single-cell images extracted from 966 images of Pap smear slides. The images are expertly classified into five categories based on the Bethesda System: Dysplastic (813), Koilocytotic (825), Metaplastic (793), Parabasal (787), and Superficial/Intermediate (831). Its larger size and diverse cell morphology make it a robust benchmark for evaluating the generalization capabilities of classifiers.

ComparisonDetector. This dataset targets object-level detection of cervical cells and clumps from cytology slides. It comprises 7,410 cervical microscopic images cropped from whole slide images (WSIs) scanned using a Pannoramic MIDI II digital slide scanner. The dataset is annotated with 48,587 object instance bounding boxes across 11 diagnostic categories, including ASC-US, ASC-H, LSIL, HSIL, SCC, AGC, Trichomonas, Candida, Flora, Herpes, and Actinomyces. The images are derived from specimens prepared

using ThinPrep methods and stained with Papanicolaou stain, conforming to the Bethesda System classification. For benchmarking, the dataset is split into 6,666 training images and 744 test images.

ISBI 2014. The effective segmentation of cervical cytology images is crucial for enhancing diagnostic accuracy and facilitating targeted interventions. To evaluate the performance of UniCAS on segmentation tasks, we utilize a publicly available dataset from the ISBI 2014 Overlapping Cervical Cytology Image Segmentation Challenge.[38,50] This dataset comprises 945 image regions, each with a size of 512 × 512 pixels, and is annotated with dual boundaries for nuclei and cytoplasm. Following the guidelines of the challenge, the dataset is organized into three subsets: 45 for training, 90 for validation, and 810 for testing.

CNSeg. This dataset is a public benchmark for cervical nuclear boundary delineation, featuring images captured at a clinical-grade 40× magnification. It is structured with a robust training/validation split, containing 3,010 training images with 74,759 expert annotations and 477 test images with 11,123 annotations, all at a standard 512 × 512 pixel resolution.

Cx22-multi. This public dataset consists of 500 cytology images, divided into 400 for training and 100 for testing. The training set contains 2,910 cytoplasm clumps, which correspond to 4,537 individual cytoplasm instances, while the test set includes 829 clumps corresponding to 1,296 instances. All images are 512 × 512 pixels and come with full annotations for multi-cell cytoplasm segmentation.

METHOD DETAILS

Pre-training of UniCAS encoder

For UniCAS encoder training, we employ DINOv2[26] that leverages student-teacher knowledge distillation for pre-training large ViT models. Our training methodology exploits multi-scale representation learning, which is critical for capturing both cellular-level details and contextual tissue organization in cytology specimens. From each patch (256 × 256 pixels) extracted from a WSI, we create multiple augmented crops at different scales: two 224 × 224 global crops that preserve most of the original patch content, eight 96 × 96 local crops that focus on smaller cellular patches potentially containing diagnostic features, and additional masked crops where random parts inside the global crops are masked. For local crops, the student network aims to produce feature representations that match those generated by the teacher network from global crops. For masked crops, the student network performs a reconstruction task, predicting the masked regions based on the surrounding non-masked visible context. The teacher network parameters are updated using an Exponential Moving Average (EMA) of the student network parameters, creating a more stable learning target.

This multi-scale approach enables the model to simultaneously learn holistic contextual patterns from larger regions while capturing fine-grained morphological features from smaller cellular structures. In our implementation, the scaling range for global crops is set between 0.5 and 1.0, and the initial learning rate is established at 3×10^{-4}. Training is conducted on 8 NVIDIA A100 GPUs with a batch size of 768, totaling one million training steps to ensure comprehensive feature learning across our diverse cytology dataset. To further enhance robustness against the inherent staining variability in cytology specimens, each crop undergoes random color augmentations, brightness adjustments, and contrast variations, which are used to mimic the staining inconsistencies encountered in real clinical settings.

Multi-task aggregator for slide-level diagnosis

Current slide-level diagnosis models are primarily limited to single-task applications.[51–53] However, such single-task paradigms face critical limitations in clinical practice, as the sequential processing of multiple diagnostic tasks through separate models leads to prohibitive computational costs and significant time delays. To address these limitations and streamline the diagnostic workflow, we propose the multi-task aggregator capable of conducting concurrent slide-level diagnostics (Figure S2).

Our multi-task aggregator begins by collecting patch classification (CLS) tokens from the UniCAS encoder, with each WSI yielding hundreds to thousands of tokens representing local patch features, and we treat these CLS tokens as the patch tokens. In addition to these CLS tokens, we introduce dedicated task tokens, where each task token is responsible for solving a specific slide-level diagnostic task, such as cancer screening, candidiasis testing, and clue cell diagnosis. These task tokens dynamically aggregate task-specific features through Task-specific Attention, working in tandem with patch CLS tokens. The integrated tokens then undergo progressive feature refinement through a series of multi-task transformer blocks that incorporate a Task Mixture of Experts (MoE) mechanism, enabling diagnostic tasks to develop task-specific feature transformations without interference, addressing the optimization challenges common in multi-task learning scenarios. Ultimately, the refined task representations are channeled into specialized decision heads to generate final diagnostic outcomes with maintained task specificity.

Task-specific attention

In WSIs, patches that contain positive diagnostic cues are typically in the minority, while most represent negative or irrelevant backgrounds.[54,55] These sparse but pathologically significant cellular features are critical for accurate diagnosis, as they contain the key evidence needed to identify abnormalities. Based on this observation, we introduce Task-specific Attention, where each task token evaluates and selects the top-m most significant patch tokens that contribute strongly to the specific diagnostic task under consideration.

As shown in the bottom-left part of Figure S2, we assign a query vector $Q_i \in R^{(n \times c)}$ ($i = 1, \cdots, t$) to the i-th task token for t different tasks. Initially, the key matrix $K \in R^{n \times c}$ and value matrix $V \in R^{n \times c}$ are derived from the n patch tokens and shared across all tasks. The vector Q_i

is utilized to compute masked attention scores (MS_i) with K, after which only the top-m most significant attention weights are retained. This mechanism can be described by the following equation:

$$MS_i = \mathrm{Softmax}\left(\mathrm{Top}_m\left(\frac{Q_i \cdot K^T}{\sqrt{c}}\right)\right), \quad \text{(Equation 1)}$$

where Top_m is an operation that filters and keeps only the m largest values while setting all others to zero ($m = 256$ in our settings, ablation study in Table S19). This selective attention mechanism effectively transforms the dense attention map into a sparse low-rank matrix, substantially reducing the computational complexity of each task and significantly enhancing overall diagnostic processing speed. The masked score MS_i is utilized to update the current task token $Q_i \in R^{1\times c}$:

$$Q_i' = MS_i \cdot V. \quad \text{(Equation 2)}$$

Concurrently, for the i-th task, patch tokens are masked using MS_i, retaining only the m selected features while zeroing all others. These masked patch tokens, which maintain their original dimension $R^{n\times c}$ despite being sparse, are concatenated with the updated task token Q_i' and processed through a task-specific projection layer ($\mathrm{Project}_i$). For each task, the projection operation transforms the concatenated tokens to dimension $R^{(1+n)\times c}$. After processing all tasks through their respective projection layers, we sum the resulting patch token representations to form a consolidated patch representation of size $R^{n\times c}$. This consolidated patch representation is then concatenated with all t task tokens to produce the final representation $X \in R^{(t+n)\times c}$.

Task MoE

In multi-task learning scenarios, the conventional feedforward network (FFN) in ViT often struggles with task interference and optimization imbalance. To address these challenges, we incorporate a Mixture of Experts (MoE) structure[56] that effectively partitions the parameter space, allowing specialized processing for different diagnostic tasks. However, conventional MoE implementations that employ top-routing strategies are suboptimal for our framework, as they would introduce an additional discrete selection process on top of our Task-specific Attention mechanism, potentially exacerbating load imbalances among experts. Instead, we adopt a soft MoE architecture,[57] which computes weighted averages of tokens and processes each with its respective expert, enabling smoother parameter updates and better task specialization.

Our Task MoE implementation applies dual softmax operations for slot construction and output combination, creating fully differentiable pathways that facilitate end-to-end learning while maintaining balanced expert utilization and preserving task-specific feature transformations. As illustrated in Figure S2 (lower right), we construct s slots, which are weighted combinations of input tokens where each slot is processed by a dedicated expert. Given input tokens $X \in R^{(t+n)\times c}$, we establish a per-slot relevance score map via a learnable projection matrix $W_s \in R^{c\times s}$, where s represents the number of slots, and it is set to 8 in our implementation (ablation study in Table S20). The token weight matrix $W_t \in R^{(t+n)\times s}$ quantifying the contribution of each token to individual slots is derived by calculating the softmax column-wise of the relevance score map. Simultaneously, the expert weight matrix W_e is derived by applying row-wise softmax to the same relevance score map along the slot dimension. The slot matrix $S \in R^{s\times c}$ is synthesized as:

$$S = W_t^{\top} \cdot X. \quad \text{(Equation 3)}$$

Here, each slot S_j (each row in the matrix S) represents a weighted ensemble of all input tokens, and each expert E_j ($j = 1,\cdots,s$) processes its assigned slot S_j, resulting in $\tilde{Y} \in R^{s\times c}$:

$$\tilde{Y} = [E_1(S_1); E_2(S_2); \cdots; E_s(S_s)] \quad \text{(Equation 4)}$$

The final output $Y \in R^{(t+n)\times c}$ is obtained through the weighted combination of expert outputs, formulated as:

$$Y = W_e \cdot \tilde{Y}. \quad \text{(Equation 5)}$$

The Task-specific Attention and Task MoE together form a processing block that is repeated L times ($L = 8$ in our implementation), progressively refining task-specific features through multiple layers of feature transformation. After the final block, the first t tokens in the output Y correspond to the t task tokens, each representing a specific diagnostic task, and these task tokens are passed through task-specific decision heads to generate the final diagnostic predictions. For model training, we use the Adam optimizer with a batch size of 16, a learning rate of 5×10^{-5}, and a cosine scheduler. The model that performs best on the validation dataset is ultimately used for testing.

Region-level analysis

For region-level classification, we utilize Linear Probing,[58] which provides an intuitive demonstration of the encoder's performance. We process the CLS token extracted from each patch through a simple two-layer linear network to map the token and generate outputs for the target classification task. This lightweight approach enables rapid evaluation of region-level classification performance while preserving the rich feature representations learned by the pre-trained encoders. In our implementation, the dimension of the hidden layer is set to 768, and these classification models are trained for 30 epochs using the Adam optimizer with a batch size of 32. Learning rate is set to 1×10^{-4} managed by the cosine scheduler and early stopping strategy.

For both detection and segmentation, we employ ViTDet,[31] which seamlessly integrates pre-trained ViT models like UniCAS into the Mask R-CNN[59] framework while preserving the original architecture. The ViT-style encoder produces feature embeddings at a size of $1/16 \times 1/16$ of the original input image. To create a feature pyramid compatible with Mask R-CNN's multi-scale architecture, we implement a feature transformation process. This process involves bidirectional scaling: downsampling the 1/16-size base features to 1/32 size using strided convolutions, while simultaneously upsampling to 1/8 and 1/4 sizes through transposed convolutions. The resulting multi-scale feature representation (spanning 1/32, 1/16, 1/8, and 1/4 sizes) feeds into Mask R-CNN's Feature Pyramid Network (FPN), enabling the detection and segmentation heads to operate across multiple scales. This approach preserves fine-grained cellular details while capturing broader contextual information critical for accurate cervical cytology analysis. The detection and segmentation models are trained for 100,000 steps with a learning rate of 1×10^{-4}. We use a batch size of 2 for 1024-resolution inputs and 4 for 512-resolution inputs.

Pixel-level enhancement

Beyond recognition capabilities, we explore UniCAS's potential in pixel-level image enhancement, an application rarely investigated for foundation models in computational pathology. We hypothesize that features extracted by UniCAS can guide enhancement networks to preserve semantic consistency, preventing artifacts or distortions in the enhanced output. While foundation models like UniCAS are typically trained on high-quality images, potentially limiting their effectiveness when extracting features from degraded inputs, our experiments demonstrate this concern is unfounded in practice. To ensure fair comparison between different foundation models for image enhancement, we adopt the DA-CLIP framework.[33] DA-CLIP is a diffusion-based enhancement framework that leverages CLIP's semantic understanding capabilities to guide the restoration process of degraded images. In this approach, an image encoder extracts semantic information from input images to guide the enhancement of degraded images through a diffusion model, integrating these features via cross-attention mechanisms to preserve important diagnostic details. For fair evaluation across various foundation models, we replace the original image encoder in DA-CLIP with each respective foundation model under consideration, followed by fine-tuning to optimize the performance of the complete diffusion-based enhancement system. This fine-tuning is conducted for 100,000 steps on an NVIDIA A100 GPU. We employ the Adam optimizer with a batch size of 8 and an initial learning rate of 1×10^{-4}.

QUANTIFICATION AND STATISTICAL ANALYSIS

All statistical analyses are performed using Python 3.11. Performance metrics for classification tasks, including accuracy, AUC, sensitivity, specificity, and F1-score, are computed using the torchmetrics library. For region-level detection, Average Precision metrics (AP, AP50, AP75, and APm) are calculated using the pycocotools library to evaluate performance across various IoU thresholds. For region-level segmentation, we report AP, Dice score, and F1-score. For pixel-level enhancement, image fidelity and perceptual quality are assessed using the SSIM, PSNR, and LPIPS. To ensure robust and reliable evaluation, 95% confidence intervals are calculated for all reported metrics. For experiments with pre-defined train-test splits, CIs are generated using bootstrap resampling with 1,000 iterations on the test set predictions. For experiments involving cross-validation, CIs are derived by aggregating results across all folds.

Cell Reports Medicine, Volume 7

Supplemental information

UniCAS: A foundation model

for cervical cytology screening

Haotian Jiang, Jiangdong Cai, Zhenrong Shen, Mengjie Xu, Manman Fei, Haolin Huang, Xinyu Wang, Rui Bi, Dinggang Shen, Lichi Zhang, and Qian Wang

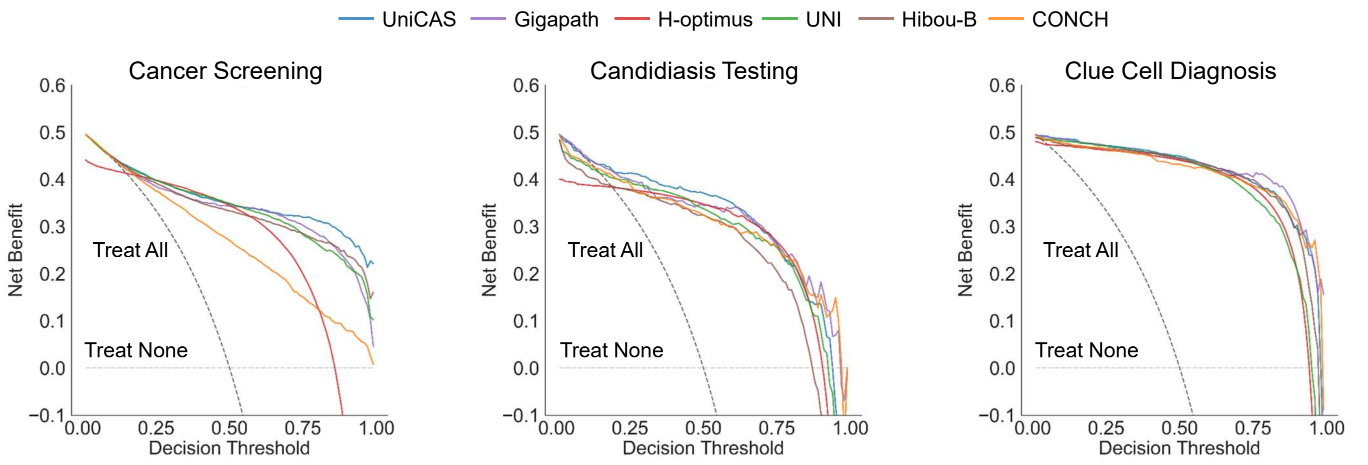

Figure S1. Decision curve for slide-level diagnosis. A model is considered clinically useful if its curve lies above both the "Treat All" and "Treat None" strategies. The curve that lies above others indicates the greatest net benefit. Related to Figure 2.

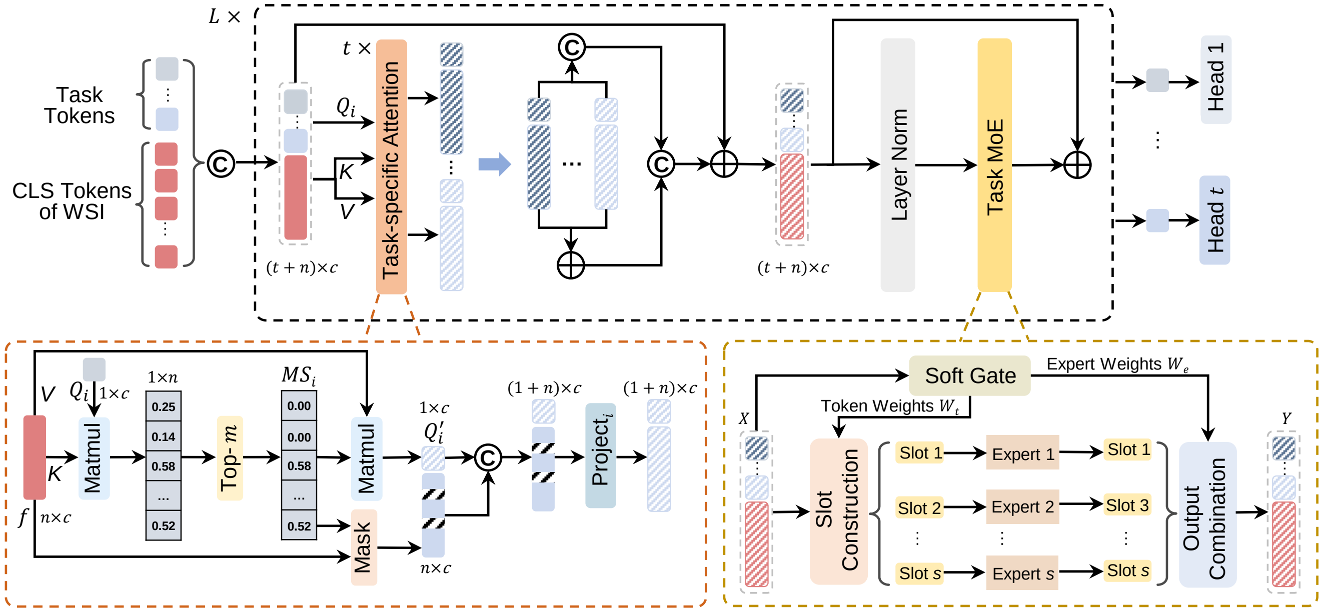

Figure S2. Overview of the multi-task aggregator (top), with Task-specific Attention and Task Mixture of Experts (MoE) zoomed in (bottom). Related to STAR Methods.

Table S1. Age distribution of pre-training data. Related to Figure 1.

**Ages**	**Number of Slides**
15–20	296
21–25	1,635
26–30	3,758
31–35	7,729
36–40	8,024
41–45	6,936
46–50	6,112
51–55	5,827
56–60	4,217
61–65	2,022
66–70	1,174
71–75	545
76–80	193
81–85	50
86–90	14
Total	48,532

Table S2. Comparison of slide-level diagnosis results using TransMIL (%, 95% CI). Related to Figure 2.

Task	Encoder	AUC	F1-score	Sensitivity	Specificity
Cancer Screening	Gigapath	90.32 (89.53–91.11)	82.96 (81.95–83.98)	81.58 (80.15–83.01)	84.68 (83.41–85.95)
	H-optimus	89.86 (89.06–90.66)	82.56 (81.56–83.57)	82.93 (81.51–84.35)	82.14 (80.78–83.49)
	UNI	89.96 (89.18–90.75)	82.50 (81.38–83.62)	80.41 (78.86–81.96)	**85.45 (84.19–86.71)**
	Hibou-B	88.40 (87.55–89.24)	80.81 (79.69–81.92)	80.44 (79.09–81.79)	81.40 (80.10–82.71)
	CONCH	82.35 (81.31–83.39)	75.12 (73.96–76.28)	75.03 (73.44–76.61)	75.25 (73.61–76.88)
	**UniCAS**	**90.90 (90.12–91.68)**	**83.70 (82.65–84.75)**	**83.37 (82.01–84.73)**	84.10 (82.79–85.40)
Candidiasis Testing	Gigapath	90.77 (87.40–94.14)	83.12 (78.69–87.54)	82.48 (76.58–88.39)	83.88 (78.00–89.76)
	H-optimus	90.26 (86.84–93.68)	83.29 (78.85–87.73)	**83.16 (76.92–89.40)**	83.54 (77.78–89.31)
	UNI	90.44 (86.99–93.88)	82.34 (77.73–86.96)	78.17 (71.43–84.91)	89.26 (84.29–94.23)
	Hibou-B	87.02 (83.18–90.86)	76.96 (71.16–82.76)	72.77 (65.79–79.75)	84.14 (78.23–90.06)
	CONCH	87.18 (83.35–91.00)	79.98 (74.81–85.15)	77.98 (71.63–84.34)	83.28 (77.51–89.04)
	**UniCAS**	**91.70 (88.51–94.90)**	**84.72 (80.31–89.13)**	81.46 (75.52–87.40)	**89.79 (85.09–94.49)**
Clue Cell Diagnosis	Gigapath	97.74 (96.74–98.73)	93.56 (91.68–95.44)	94.30 (91.88–96.71)	92.44 (89.91–94.97)
	H-optimus	97.30 (96.26–98.34)	92.84 (90.91–94.78)	**94.44 (92.05–96.83)**	91.08 (88.09–94.08)
	UNI	97.64 (96.65–98.63)	93.02 (91.08–94.95)	93.53 (90.96–96.10)	92.28 (89.47–95.10)
	Hibou-B	97.48 (96.29–98.68)	94.18 (92.46–95.90)	93.96 (91.45–96.47)	94.58 (92.27–96.90)
	CONCH	96.90 (95.81–97.99)	91.62 (89.52–93.73)	91.40 (88.43–94.38)	92.08 (89.29–94.86)
	**UniCAS**	**97.81 (96.73–98.89)**	**94.24 (92.52–95.96)**	93.84 (91.28–96.39)	**94.61 (92.31–96.91)**

Table S3. Comparison of slide-level diagnosis results using DTFree (%, 95% CI). Related to Figure 2.

Task	Encoder	AUC	F1-score	Sensitivity	Specificity
Cancer Screening	Gigapath	92.00 (91.36–92.64)	85.17 (84.17–86.17)	82.00 (80.57–83.44)	89.44 (88.30–90.59)
	H-optimus	91.64 (90.93–92.36)	83.52 (82.44–84.59)	**83.52 (82.19–84.86)**	83.32 (81.89–84.74)
	UNI	90.50 (89.73–91.28)	82.38 (81.28–83.47)	80.20 (78.73–81.68)	85.62 (84.28–86.97)
	Hibou-B	91.02 (90.26–91.79)	83.02 (81.96–84.08)	81.51 (80.06–82.95)	85.10 (83.75–86.45)
	CONCH	88.71 (87.83–89.59)	80.82 (79.69–81.94)	81.39 (80.01–82.78)	80.14 (78.73–81.55)
	**UniCAS**	**92.33 (91.61–93.05)**	**86.24 (85.30–87.17)**	83.24 (81.86–84.63)	**90.20 (89.08–91.31)**
Candidiasis Testing	Gigapath	90.76 (87.50–94.02)	83.72 (79.18–88.27)	**83.92 (78.39–89.44)**	83.84 (78.12–89.57)
	H-optimus	90.80 (87.59–94.02)	82.50 (77.86–87.13)	80.49 (74.61–86.36)	85.08 (79.31–90.85)
	UNI	91.02 (87.71–94.32)	82.20 (77.49–86.92)	73.70 (66.89–80.50)	**95.22 (91.82–98.63)**
	Hibou-B	89.54 (85.99–93.10)	81.12 (75.99–86.25)	75.25 (68.32–82.17)	89.84 (84.93–94.74)
	CONCH	88.56 (84.73–92.38)	80.58 (75.71–85.44)	79.20 (73.05–85.34)	82.07 (75.68–88.46)
	**UniCAS**	**92.26 (89.41–95.12)**	**85.67 (81.48–89.86)**	81.86 (76.14–87.59)	90.50 (85.99–95.00)
Clue Cell Diagnosis	Gigapath	97.74 (96.72–98.76)	93.37 (91.42–95.32)	92.73 (90.06–95.41)	94.11 (91.67–96.55)
	H-optimus	97.64 (96.67–98.61)	93.37 (91.49–95.25)	92.52 (89.83–95.22)	94.19 (91.78–96.60)
	UNI	98.12 (97.27–98.97)	**93.90 (92.06–95.75)**	92.12 (89.43–94.81)	**95.79 (93.80–97.78)**
	Hibou-B	97.86 (96.83–98.88)	93.82 (92.04–95.59)	92.78 (90.08–95.49)	95.02 (92.66–97.37)
	CONCH	97.38 (96.40–98.37)	91.48 (89.53–93.42)	92.11 (89.36–94.86)	90.46 (87.50–93.43)
	**UniCAS**	**98.20 (97.31–99.10)**	93.84 (91.94–95.73)	**93.41 (90.81–96.01)**	94.51 (92.26–96.76)

Table S4. Class-wise metrics for slide-level fine-grained subtype diagnosis (%, 95%CI). Related to Figure 2.

Class	Encoder	Sensitivity	AUC	F1-score	Specificity
NILM	Gigapath	42.31 (26.47–58.14)	80.05 (72.42–87.69)	51.23 (35.80–66.67)	**94.57 (91.08–98.05)**
	H-optimus	72.35 (58.33–86.37)	83.22 (75.87–90.57)	59.55 (47.91–71.19)	82.47 (76.62–88.31)
	UNI	64.07 (48.65–79.49)	81.47 (73.59–89.36)	53.08 (40.68–65.49)	81.05 (75.15–86.96)
	Hibou-B	55.80 (40.47–71.12)	80.65 (73.26–88.05)	55.69 (43.08–68.30)	89.24 (84.35–94.12)
	CONCH	35.56 (20.00–51.11)	71.67 (63.34–80.00)	35.77 (21.54–50.00)	85.19 (79.58–90.80)
	**UniCAS**	**74.46 (61.11–87.81)**	**90.32 (86.16–94.49)**	**67.46 (56.41–78.51)**	88.32 (83.43–93.20)
ASC-US	Gigapath	22.19 (8.82–35.56)	**65.42 (55.58–75.26)**	24.54 (10.71–38.36)	85.60 (79.87–91.33)
	H-optimus	20.61 (7.89–33.33)	62.23 (53.45–71.01)	23.83 (10.17–37.50)	**88.13 (83.11–93.16)**
	UNI	18.85 (7.14–30.56)	61.91 (52.35–71.46)	21.23 (8.70–33.77)	86.08 (80.64–91.52)
	Hibou-B	**33.27 (18.92–47.62)**	61.84 (52.46–71.22)	**32.48 (19.17–45.78)**	82.89 (77.24–88.54)
	CONCH	18.09 (6.38–29.79)	48.68 (39.19–58.17)	17.49 (6.06–28.92)	78.40 (71.85–84.94)
	**UniCAS**	28.60 (14.71–42.50)	61.35 (51.92–70.78)	31.29 (17.24–45.33)	86.23 (80.79–91.67)
LSIL	Gigapath	**54.68 (38.76–70.59)**	72.85 (64.99–80.71)	**43.06 (30.76–55.36)**	74.95 (68.15–81.76)
	H-optimus	40.41 (25.00–55.81)	73.19 (65.65–80.73)	37.17 (24.32–50.01)	81.12 (75.00–87.25)
	UNI	43.45 (28.56–58.34)	71.38 (63.73–79.03)	40.75 (28.17–53.34)	82.94 (76.92–88.96)
	Hibou-B	38.02 (23.40–52.64)	71.99 (63.36–80.62)	38.28 (25.00–51.55)	**84.82 (79.22–90.41)**
	CONCH	38.25 (22.22–54.29)	73.38 (65.91–80.85)	35.21 (21.88–48.55)	81.09 (75.31–86.88)
	**UniCAS**	45.04 (30.56–59.52)	**73.87 (66.06–81.67)**	41.83 (28.92–54.74)	82.39 (76.47–88.31)
ASC-H	Gigapath	**45.13 (29.73–60.53)**	**71.05 (62.53–79.57)**	39.26 (26.67–51.86)	77.78 (71.52–84.05)
	H-optimus	25.21 (12.19–38.24)	69.55 (60.54–78.56)	26.90 (14.29–39.51)	84.71 (78.85–90.57)
	UNI	33.11 (18.60–47.62)	70.06 (59.75–80.38)	33.29 (20.00–46.58)	84.34 (78.81–89.86)
	Hibou-B	32.89 (18.42–47.37)	58.74 (47.50–69.97)	30.63 (17.50–43.75)	79.60 (73.29–85.90)
	CONCH	36.47 (21.43–51.52)	65.96 (56.59–75.34)	35.92 (22.49–49.35)	83.70 (77.92–89.48)
	**UniCAS**	40.87 (25.64–56.11)	66.24 (55.73–76.75)	**41.43 (27.78–55.07)**	**86.60 (81.17–92.03)**
HSIL	Gigapath	36.47 (20.00–52.95)	79.32 (70.88–87.76)	39.78 (24.00–55.56)	**91.07 (86.67–95.48)**
	H-optimus	**50.45 (33.33–67.57)**	79.77 (70.80–88.74)	**48.72 (34.28–63.16)**	88.84 (84.17–93.51)
	UNI	47.51 (30.30–64.71)	**81.79 (74.07–89.50)**	48.46 (33.96–62.96)	90.53 (86.06–95.00)
	Hibou-B	44.98 (28.20–61.76)	75.96 (67.43–84.48)	43.31 (29.03–57.58)	87.36 (82.17–92.55)
	CONCH	35.23 (19.35–51.11)	74.43 (65.07–83.80)	33.67 (19.44–47.89)	85.68 (80.11–91.25)
	**UniCAS**	46.87 (30.76–62.97)	80.98 (72.89–89.08)	46.74 (32.14–61.34)	89.13 (84.18–94.08)
Avg.	Gigapath	40.14 (33.36–46.91)	73.93 (69.52–78.34)	40.15 (33.16–47.15)	84.99 (83.25–86.73)
	H-optimus	41.43 (35.09–47.78)	73.80 (69.45–78.15)	39.48 (32.97–45.98)	85.26 (83.54–86.98)
	UNI	41.03 (34.37–47.70)	73.79 (69.61–77.97)	39.70 (32.92–46.49)	85.23 (83.54–86.92)
	Hibou-B	40.55 (33.78–47.31)	70.28 (65.56–75.00)	40.33 (33.89–46.78)	85.11 (83.45–86.77)
	CONCH	32.09 (25.52–38.66)	66.89 (62.49–71.29)	31.69 (25.22–38.16)	82.95 (81.29–84.62)
	**UniCAS**	**47.21 (40.73–53.69)**	**74.63 (70.18–79.09)**	**46.13 (39.80–52.46)**	**86.77 (85.11–88.42)**

Table S5. Comparison of region-level diagnoses for cervical abnormality classification (%, 95% CI). Related to Figure 3.

Encoder	ACC (Macro)	F1-score (Macro)	Sensitivity	Specificity
Gigapath	93.40 (93.14–93.67)	91.15 (90.80–91.50)	97.82 (97.51–98.13)	88.98 (88.57–89.38)
H-optimus	93.24 (92.99–93.50)	89.95 (89.58–90.32)	**99.00 (98.79–99.20)**	87.47 (87.02–87.92)
UNI	92.86 (92.59–93.13)	89.46 (89.09–89.84)	98.79 (98.56–99.02)	86.91 (86.43–87.39)
Hibou-B	92.72 (92.43–93.00)	89.76 (89.38–90.15)	97.41 (97.10–97.72)	88.00 (87.54–88.47)
CONCH	86.53 (86.22–86.85)	80.61 (80.14–81.08)	97.03 (96.82–97.24)	76.03 (75.39–76.64)
**UniCAS**	**94.23 (94.00–94.46)**	**91.47 (91.13–91.81)**	98.84 (98.62–99.07)	**89.63 (89.20–90.06)**

Table S6. Comparison of region-level diagnoses for candidiasis classification (%, 95% CI). Related to Figure 3.

Encoder	ACC	F1-score	Sensitivity	Specificity
Gigapath	95.53 (94.72–96.34)	95.45 (94.37–96.53)	92.55 (88.80–96.30)	**98.48 (96.96–100.0)**
H-optimus	95.62 (94.95–96.29)	95.59 (94.83–96.35)	94.42 (91.86–96.98)	96.83 (94.63–99.03)
UNI	95.82 (95.44–96.20)	95.84 (95.22–96.46)	94.76 (91.22–98.30)	96.78 (93.57–100.0)
Hibou-B	95.69 (95.08–96.30)	95.51 (94.85–96.17)	94.38 (91.55–97.21)	97.00 (94.55–99.45)
CONCH	93.79 (92.85–94.73)	93.64 (92.83–94.45)	92.05 (89.00–95.10)	95.62 (91.92–99.32)
**UniCAS**	**96.15 (95.82–96.48)**	**96.30 (95.86–96.74)**	**95.68 (94.25–97.11)**	96.62 (95.63–97.61)

Table S7. Comparison of few-shot experiments on clue cell classification (F1-score%, 95% CI). Related to Figure 3.

Encoder	K=1	K=2	K=4	K=8	K=16
Gigapath	**74.5 (71.9–77.1)**	78.6 (76.0–81.2)	86.1 (83.9–88.0)	95.2 (94.0–96.6)	97.3 (96.2–98.3)
H-optimus	66.6 (63.6–69.6)	66.3 (63.4–69.2)	89.8 (88.0–91.7)	92.5 (90.7–94.0)	96.7 (95.5–97.7)
UNI	68.1 (65.2–71.1)	67.2 (64.1–70.3)	85.9 (83.8–88.0)	85.1 (82.8–87.6)	96.4 (95.2–97.5)
Hibou-B	**74.5 (71.9–77.3)**	80.7 (78.3–83.0)	87.1 (85.2–89.2)	94.0 (93.0–95.0)	94.6 (93.2–96.0)
CONCH	50.1 (46.8–53.1)	60.0 (56.7–63.1)	68.1 (65.0–71.0)	73.3 (70.5–76.0)	84.3 (82.0–86.5)
**UniCAS**	**74.5 (71.8–77.1)**	**84.3 (81.8–86.6)**	**91.7 (90.0–93.4)**	**96.7 (95.5–97.7)**	**98.4 (97.6–99.1)**

Table S8. Region-level cervical abnormality grading results (95% CI). Related to Figure 3.

Encoder	Top-1 ACC	Top-2 ACC	Sensitivity	F1-score
Gigapath	67.97 (67.36–68.58)	93.25 (92.86–93.64)	59.88 (59.18–60.58)	58.18 (56.79–59.57)
H-optimus	67.97 (67.01–68.93)	92.98 (92.21–93.75)	61.10 (59.58–62.62)	59.06 (55.56–62.56)
UNI	67.49 (66.76–68.22)	93.27 (92.89–93.65)	61.33 (60.94–61.72)	60.83 (60.42–61.24)
Hibou-B	66.37 (65.59–67.15)	92.09 (91.69–92.49)	61.95 (60.66–63.24)	62.31 (61.55–63.07)
CONCH	65.34 (64.75–65.93)	91.76 (91.46–92.06)	58.69 (58.14–59.24)	59.02 (58.35–59.69)
**UniCAS**	**70.25 (69.51–70.99)**	**94.93 (94.61–95.25)**	**69.71 (68.93–70.49)**	**67.08 (66.08–68.08)**

Table S9. Comparison of region-level abnormal cervical cell detection (95% CI). Related to Figure 4.

Encoder	AP	AP50	AP75	APm
Gigapath	28.65 (26.46–30.84)	58.49 (54.78–62.19)	25.23 (21.91–28.55)	31.91 (28.22–35.60)
H-optimus	15.35 (13.84–16.86)	42.09 (38.62–45.55)	7.25 (5.58–8.92)	13.59 (11.23–15.95)
UNI	26.65 (24.69–28.61)	58.37 (54.91–61.83)	21.81 (18.81–24.81)	30.25 (26.53–33.96)
Hibou-B	10.76 (9.42–12.09)	29.16 (25.87–32.44)	4.98 (3.67–6.29)	7.75 (5.55–9.95)
CONCH	25.55 (23.41–27.68)	52.06 (48.53–55.58)	22.28 (19.28–25.27)	29.12 (25.70–32.53)
**UniCAS**	**29.99 (27.80–32.17)**	**62.67 (59.32–66.01)**	**26.48 (23.13–29.82)**	**34.65 (30.74–38.55)**

Table S10. Comparison of region-level clue cell detection (95% CI). Related to Figure 4.

Encoder	AP	AP50	AP75	APm
Gigapath	27.80 (24.02–31.57)	56.74 (49.91–63.57)	25.10 (20.02–30.17)	28.44 (24.10–32.77)
H-optimus	20.01 (17.20–22.82)	51.96 (46.04–57.87)	8.91 (6.27–11.55)	17.02 (13.98–20.05)
UNI	26.40 (22.89–29.90)	56.77 (51.09–62.44)	20.78 (15.92–25.63)	27.55 (22.97–32.13)
Hibou-B	22.35 (19.34–25.35)	51.67 (45.36–57.97)	14.84 (10.98–18.70)	23.74 (19.96–27.52)
CONCH	26.95 (22.93–30.96)	55.67 (49.13–62.21)	22.10 (16.79–27.41)	29.48 (25.07–33.89)
**UniCAS**	**30.06 (26.23–33.88)**	**60.48 (54.43–66.52)**	**26.27 (20.74–31.79)**	**30.05 (25.83–34.26)**

Table S11. Comparison of region-level fine-grained subtype detection (AP, 95% CI). Related to Figure 4.

Encoder	ASC-US	LSIL	ASC-H	HSIL	Avg.
Gigapath	8.76 (6.96–10.55)	18.27 (15.27–21.26)	15.41 (12.91–17.90)	7.15 (4.98–9.31)	12.47 (11.41–13.52)
H-optimus	6.14 (4.62–7.66)	11.48 (9.20–13.76)	12.11 (9.67–14.55)	5.30 (3.31–7.29)	8.73 (7.81–9.65)
UNI	10.86 (8.49–13.22)	19.17 (16.06–22.27)	15.26 (12.50–18.02)	10.08 (6.52–13.64)	13.87 (12.49–15.24)
Hibou-B	2.27 (1.46–3.07)	4.73 (3.36–6.09)	8.59 (6.75–10.42)	1.59 (0.82–2.36)	4.23 (3.61–4.85)
CONCH	6.75 (5.16–8.33)	13.82 (11.36–16.27)	13.55 (10.90–16.20)	5.00 (3.25–6.74)	9.76 (8.87–10.65)
**UniCAS**	**12.40 (9.65–15.15)**	**21.06 (17.46–24.65)**	**17.87 (14.88–20.85)**	**14.75 (10.15–19.34)**	**16.62 (14.98–18.25)**

Table S12. Comparison of region-level fine-grained subtype detection (AP50, 95% CI). Related to Figure 4.

Encoder	ASC-US	LSIL	ASC-H	HSIL	Avg.
Gigapath	19.38 (15.06–23.69)	36.16 (30.72–41.60)	30.17 (25.92–34.42)	12.81 (9.08–16.54)	24.78 (22.81–26.74)
H-optimus	16.32 (12.43–20.20)	25.81 (21.40–30.22)	26.25 (21.56–30.93)	11.43 (7.52–15.34)	19.92 (18.11–21.73)
UNI	22.72 (18.14–27.30)	39.23 (33.74–44.71)	28.39 (23.87–32.90)	18.96 (13.18–24.74)	27.45 (25.13–29.77)
Hibou-B	6.89 (4.60–9.18)	14.17 (10.51–17.83)	20.18 (16.17–24.18)	4.28 (2.19–6.37)	11.19 (9.73–12.64)
CONCH	15.80 (12.54–19.06)	31.95 (26.81–37.08)	26.29 (21.64–30.94)	10.40 (7.25–13.54)	21.23 (19.64–22.81)
**UniCAS**	**24.73 (19.73–29.73)**	**42.54 (36.50–48.57)**	**30.57 (25.81–35.32)**	**26.56 (19.14–33.97)**	**31.43 (28.72–34.14)**

Table S13. Comparison of pixel-level cervical image enhancement (95% CI). Related to Figure 5.

Encoder	SSIM↑ (%)	PSNR↑	LPIPS↓
Gigapath	74.23 (60.22–88.24)	**26.92 (20.78–33.06)**	0.1340 (0.0764–0.1916)
H-optimus	72.92 (57.87–87.97)	26.59 (20.51–32.67)	0.1353 (0.0784–0.1922)
UNI	73.20 (58.37–88.03)	26.67 (20.38–32.96)	0.1392 (0.0816–0.1968)
Hibou-B	72.04 (56.41–87.67)	26.44 (20.19–32.69)	0.1401 (0.0802–0.2000)
CONCH	72.67 (57.48–87.86)	26.68 (20.38–32.98)	0.1343 (0.0780–0.1906)
**UniCAS**	**74.45 (60.30–88.60)**	**26.92 (20.66–33.18)**	**0.1332 (0.0762–0.1902)**

Table S14. External validation on the BMT dataset(%, 95% CI). Related to Figure 6.

Class	Encoder	Sensitivity	AUC	F1-score	Specificity
NILM	Gigapath	84.02 (73.17–94.87)	90.03 (85.02–95.03)	75.54 (65.93–85.15)	80.67 (72.15–89.19)
	H-optimus	60.83 (46.66–75.00)	88.74 (83.43–94.04)	62.04 (50.00–74.08)	84.04 (76.18–91.89)
	UNI	71.40 (57.50–85.29)	95.84 (92.71–98.96)	79.28 (69.08–89.47)	**96.00 (92.00–100.0)**
	Hibou-B	84.65 (74.18–95.12)	96.26 (93.72–98.81)	83.15 (74.63–91.67)	90.97 (84.88–97.06)
	CONCH	65.00 (50.00–80.00)	79.94 (71.47–88.42)	64.79 (52.50–77.08)	83.48 (75.29–91.67)
	**UniCAS**	**93.42 (86.84–100.0)**	**99.19 (98.37–100.0)**	**93.21 (87.72–98.70)**	95.83 (91.66–100.0)
LSIL	Gigapath	52.10 (36.11–68.09)	86.14 (79.69–92.59)	61.04 (46.67–75.41)	92.02 (86.48–97.56)
	H-optimus	53.37 (37.50–69.23)	83.35 (76.44–90.25)	54.07 (40.67–67.47)	79.67 (70.59–88.75)
	UNI	84.06 (73.68–94.44)	93.13 (88.83–97.43)	78.40 (69.13–87.67)	84.35 (76.19–92.50)
	Hibou-B	66.67 (51.28–82.05)	91.15 (86.32–95.98)	69.91 (58.33–81.48)	88.25 (81.33–95.18)
	CONCH	53.02 (38.30–67.74)	73.92 (64.74–83.09)	55.62 (42.42–68.82)	82.16 (74.07–90.24)
	**UniCAS**	**86.73 (76.08–97.37)**	**98.54 (97.24–99.85)**	**89.85 (82.86–96.84)**	**96.75 (93.51–100.0)**
HSIL	Gigapath	95.95 (91.89–100.0)	98.05 (96.12–99.97)	91.97 (86.07–97.87)	93.20 (87.67–98.73)
	H-optimus	88.55 (79.49–97.62)	96.18 (93.09–99.28)	82.89 (74.42–91.36)	87.30 (80.49–94.12)
	UNI	91.83 (83.66–100.0)	97.79 (95.86–99.73)	89.61 (82.67–96.55)	93.05 (87.36–98.73)
	Hibou-B	91.67 (83.33–100.0)	98.03 (96.43–99.62)	88.30 (80.95–95.65)	91.88 (86.11–97.65)
	CONCH	83.67 (72.34–95.00)	90.07 (84.56–95.58)	77.89 (68.40–87.38)	84.47 (76.54–92.41)
	**UniCAS**	**100.0 (100.0–100.0)**	**99.79 (99.59–100.0)**	**96.75 (93.51–100.0)**	**96.71 (93.42–100.0)**
Avg.	Gigapath	78.53 (71.96–85.09)	91.70 (87.94–95.45)	77.13 (69.69–84.58)	89.01 (85.33–92.68)
	H-optimus	67.79 (60.08–75.50)	89.48 (85.34–93.61)	66.94 (59.05–74.83)	83.70 (79.41–87.99)
	UNI	83.09 (76.47–89.71)	95.71 (93.13–98.29)	82.77 (76.04–89.50)	91.41 (88.04–94.78)
	Hibou-B	81.49 (74.88–88.10)	95.26 (92.67–97.84)	80.94 (74.10–87.77)	90.72 (87.27–94.17)
	CONCH	67.59 (59.88–75.29)	81.51 (75.73–87.29)	66.84 (58.82–74.87)	83.75 (79.62–87.88)
	**UniCAS**	**93.79 (89.60–97.98)**	**99.22 (98.51–99.92)**	**93.83 (89.54–98.11)**	**97.06 (94.99–99.12)**

Table S15. External validation of region-level classification (%, 95% CI). Related to Figure 6.

Dataset	Encoder	ACC	F1-score	Sensitivity	Specificity
Herlev	Gigapath	96.20 (93.48–98.91)	94.70 (91.02–98.37)	**94.96 (91.40–98.53)**	**94.86 (91.10–98.61)**
	H-optimus	94.30 (90.76–97.83)	93.10 (89.03–97.18)	94.68 (91.27–98.08)	94.81 (91.44–98.18)
	UNI	94.30 (90.76–97.83)	92.28 (88.07–96.50)	91.00 (85.57–96.43)	90.55 (85.22–95.88)
	Hibou-B	93.20 (89.67–96.74)	91.32 (86.70–95.93)	91.89 (86.92–96.86)	91.92 (87.51–96.34)
	CONCH	90.49 (86.41–94.57)	86.88 (81.17–92.59)	85.10 (79.04–91.15)	85.12 (78.37–91.88)
	**UniCAS**	**97.02 (94.57–99.46)**	**96.02 (92.78–99.26)**	94.73 (90.57–98.89)	94.50 (90.19–98.81)
SIPaKMeD	Gigapath	95.68 (94.32–97.04)	95.74 (94.38–97.10)	96.06 (94.83–97.28)	98.95 (98.62–99.28)
	H-optimus	94.88 (93.33–96.42)	94.74 (93.26–96.23)	95.23 (93.92–96.54)	98.76 (98.40–99.11)
	UNI	96.24 (94.94–97.53)	96.20 (94.92–97.47)	96.38 (95.15–97.61)	99.08 (98.76–99.41)
	Hibou-B	93.89 (92.22–95.56)	93.80 (92.22–95.38)	94.19 (92.71–95.67)	98.48 (98.08–98.89)
	CONCH	95.06 (93.70–96.42)	94.95 (93.44–96.46)	95.23 (93.82–96.64)	98.75 (98.38–99.12)
	**UniCAS**	**97.72 (96.79–98.64)**	**97.73 (96.72–98.74)**	**97.80 (96.88–98.73)**	**99.43 (99.17–99.69)**

Table S16. External validation of region-level detection on ComparisonDetector dataset (%, 95% CI). Related to Figure 6.

Encoder	mAP	AP50	AP75	APm
Gigapath	24.18 (21.43–26.94)	49.20 (46.01–52.40)	20.86 (17.12–24.61)	18.57 (12.16–25.01)
H-optimus	8.97 (7.71–10.29)	21.45 (18.81–24.09)	6.52 (4.48–8.55)	3.74 (2.56–4.91)
UNI	25.40 (22.76–28.04)	50.65 (46.84–54.47)	**23.63 (19.43–27.83)**	**20.66 (15.33–25.99)**
Hibou-B	6.29 (5.06–7.53)	16.32 (13.60–19.04)	2.72 (1.75–3.69)	2.35 (1.35–3.44)
CONCH	13.43 (11.51–15.35)	30.06 (27.22–32.93)	9.42 (7.08–11.76)	10.60 (7.13–14.07)
**UniCAS**	**26.99 (23.47–30.51)**	**53.59 (49.42–57.77)**	23.13 (18.06–28.20)	14.74 (12.50–16.98)

Table S17. External validation of region-level segmentation (%, 95% CI). Related to Figure 6.

Dataset	Encoder	AP	Dice	F1-score
ISBI 2014	Gigapath	65.60 (65.45–65.75)	90.72 (90.58–90.86)	91.79 (91.44–92.14)
	H-optimus	40.02 (39.24–40.80)	85.47 (85.30–85.63)	78.58 (77.92–79.25)
	UNI	62.33 (61.63–63.02)	91.21 (91.07–91.35)	94.04 (93.49–94.59)
	Hibou-B	19.62 (19.15–20.08)	80.93 (80.71–81.14)	43.18 (42.73–43.63)
	CONCH	57.57 (57.35–57.78)	89.83 (89.67–89.99)	88.11 (87.88–88.34)
	**UniCAS**	**69.09 (68.41–69.77)**	**92.11 (91.97–92.24)**	**96.13 (95.82–96.43)**
CNSeg	Gigapath	55.34 (54.86–55.81)	90.41 (90.38–90.43)	82.15 (81.90–82.40)
	H-optimus	43.58 (42.42–44.74)	87.57 (87.40–87.73)	63.04 (61.98–64.09)
	UNI	54.63 (54.27–54.98)	90.29 (90.27–90.31)	82.13 (81.65–82.60)
	Hibou-B	13.25 (12.64–13.85)	81.13 (80.96–81.29)	37.87 (36.97–38.77)
	CONCH	53.42 (52.05–54.78)	90.09 (89.93–90.25)	82.42 (81.59–83.25)
	**UniCAS**	**58.38 (58.28–58.47)**	**90.69 (90.64–90.74)**	**86.26 (85.63–86.88)**
Cx22-Multi	Gigapath	75.70 (74.27–77.13)	94.34 (94.12–94.56)	95.25 (94.47–96.03)
	H-optimus	60.12 (57.72–62.52)	90.50 (90.14–90.85)	87.37 (86.17–88.56)
	UNI	70.13 (68.39–71.87)	94.21 (93.98–94.43)	93.26 (92.41–94.10)
	Hibou-B	64.20 (62.28–66.12)	91.24 (90.91–91.56)	89.84 (88.72–90.95)
	CONCH	**79.52 (67.55–91.48)**	93.81 (93.58–94.04)	92.87 (91.96–93.77)
	**UniCAS**	77.71 (76.53–78.88)	**94.38 (94.16–94.59)**	**96.15 (95.37–96.92)**

Table S18. External validation on ISBI 2014 dataset (%, 95% CI). Related to Figure 6.

Encoder	mAP		Dice		F1-score	
	Cytoplasm	Nuclei	Cytoplasm	Nuclei	Cytoplasm	Nuclei
Gigapath	65.74 (64.88–66.59)	65.46 (64.71–66.21)	91.32 (90.72–91.92)	90.11 (90.08–90.14)	85.05 (84.67–85.43)	98.54 (98.15–98.92)
H-optimus	49.08 (47.89–50.26)	30.94 (30.30–31.58)	88.11 (87.87–88.34)	82.83 (82.65–83.01)	84.99 (84.26–85.72)	72.19 (71.18–73.19)
UNI	62.22 (61.05–63.39)	62.43 (61.91–62.95)	91.95 (91.70–92.20)	90.47 (90.34–90.59)	90.16 (89.34–90.98)	97.92 (97.63–98.21)
Hibou-B	37.57 (36.70–38.43)	1.67 (1.50–1.84)	84.61 (84.41–84.81)	77.25 (76.89–77.61)	80.97 (80.16–81.78)	5.39 (5.05–5.72)
CONCH	53.05 (52.87–53.22)	62.31 (61.87–62.75)	89.73 (89.51–89.95)	89.93 (89.72–90.14)	79.44 (78.96–79.92)	96.78 (96.27–97.29)
**UniCAS**	**71.33 (70.18–72.48)**	**66.88 (66.42–67.35)**	**92.99 (92.76–93.23)**	**91.23 (91.12–91.33)**	**93.63 (93.14–94.11)**	**98.64 (98.38–98.90)**

Table S19. Ablation study of Top-m in multi-task aggregator (%, 95% CI). Related to STAR Methods.

Task	Parameter	ACC	AUC	Sensitivity	Specificity
Cancer Screening	m=800	84.48 (84.01–84.95)	90.80 (90.53–91.07)	81.03 (80.20–81.86)	87.08 (85.53–88.62)
	m=512	84.73 (84.44–85.03)	91.08 (90.99–91.17)	81.60 (80.73–82.47)	**87.86 (86.91–88.81)**
	m=256	86.18 (85.77–86.60)	**93.09 (93.03–93.15)**	85.51 (85.30–85.71)	86.86 (86.07–87.68)
	m=128	**86.21 (85.54–86.89)**	92.26 (91.97–92.55)	85.94 (84.89–86.98)	86.48 (84.57–88.39)
	m=64	84.25 (83.38–85.11)	92.58 (92.38–92.78)	**86.64 (85.57–87.71)**	81.85 (79.15–84.55)
Candidiasis Testing	m=800	86.21 (84.86–87.56)	92.09 (91.27–92.92)	81.85 (79.46–84.24)	**90.57 (88.36–92.47)**
	m=512	**87.14 (85.86–88.41)**	93.00 (92.00–93.99)	85.42 (81.36–89.49)	88.85 (84.31–93.39)
	m=256	86.96 (85.99–87.94)	**93.21 (92.50–93.92)**	85.71 (84.80–86.63)	88.21 (86.50–89.93)
	m=128	86.13 (85.93–86.32)	92.45 (92.27–92.63)	86.18 (83.68–88.67)	86.07 (83.23–88.91)
	m=64	84.64 (84.11–85.17)	92.75 (92.20–93.29)	**86.96 (84.48–89.43)**	82.33 (81.46–83.20)
Clue Cell Diagnosis	m=800	94.02 (93.75–94.30)	98.17 (97.81–98.52)	94.27 (92.36–96.21)	93.85 (92.50–95.20)
	m=512	94.25 (93.69–94.80)	98.40 (98.30–98.50)	94.63 (93.34–95.91)	93.87 (92.55–95.19)
	m=256	**95.37 (94.69–96.05)**	**98.93 (98.45–99.40)**	95.62 (94.79–96.46)	**95.11 (93.45–96.78)**
	m=128	95.13 (94.58–95.67)	98.46 (98.39–98.53)	95.93 (95.15–96.72)	94.32 (93.07–95.58)
	m=64	95.14 (94.26–96.01)	98.44 (98.38–98.50)	**96.40 (95.73–97.06)**	94.03 (92.93–95.13)

Table S20. Ablation study of the number of experts (s) in multi-task aggregator (%, 95% CI). Related to STAR Methods.

Task	Parameter	ACC	AUC	Sensitivity	Specificity
Cancer Screening	s=2	85.16 (84.02–86.30)	92.15 (91.58–92.73)	83.50 (83.22–83.78)	86.82 (84.69–88.94)
	s=4	85.58 (85.33–85.82)	92.44 (92.20–92.68)	84.58 (83.23–85.93)	86.57 (85.54–87.61)
	s=8	**86.18 (85.77–86.60)**	**93.09 (93.03–93.15)**	**85.51 (85.30–85.71)**	86.86 (86.07–87.68)
	s=12	85.94 (85.27–86.60)	92.79 (92.40–93.18)	85.45 (84.47–86.44)	86.42 (85.21–87.64)
	s=16	86.01 (85.25–86.76)	92.69 (92.44–92.95)	85.08 (84.22–85.94)	**86.93 (84.69–89.18)**
Candidiasis Testing	s=2	85.71 (84.09–87.34)	92.46 (91.81–93.10)	84.71 (82.90–86.53)	86.71 (84.32–89.11)
	s=4	86.71 (85.32–88.10)	92.88 (91.95–93.81)	86.29 (83.46–89.11)	87.17 (83.69–90.59)
	s=8	86.96 (85.99–87.94)	93.21 (92.50–93.92)	85.71 (84.80–86.63)	88.21 (86.50–89.93)
	s=12	87.02 (84.55–89.50)	93.17 (92.14–94.19)	85.90 (84.92–86.89)	88.14 (84.14–92.15)
	s=16	**88.12 (86.96–89.29)**	**93.39 (92.52–94.26)**	**87.14 (84.66–89.63)**	**89.11 (86.28–91.93)**
Clue Cell Diagnosis	s=2	94.77 (94.15–95.39)	98.54 (98.13–98.95)	94.77 (93.79–95.76)	94.76 (93.78–95.74)
	s=4	95.28 (94.21–96.34)	98.29 (97.60–98.98)	96.34 (94.52–98.17)	94.21 (93.31–95.11)
	s=8	95.37 (94.69–96.05)	**98.93 (98.45–99.40)**	95.62 (94.79–96.46)	**95.11 (93.45–96.78)**
	s=12	**95.39 (94.79–96.04)**	98.78 (98.11–99.45)	95.68 (95.44–95.91)	**95.11 (93.61–96.61)**
	s=16	95.17 (95.05–95.29)	**98.93 (98.77–99.09)**	**96.35 (96.03–96.67)**	93.99 (93.48–94.49)
